# Experimental emulation of charge air cooler temperature and pressure on an engine dynamometer for air–fuel ratio optimization and pulsation reduction

**DOI:** 10.1038/s41598-026-53715-w

**Published:** 2026-05-30

**Authors:** Rajendra Kumar Jagadeesh, P. M. G. Bashir Asdaque, Viswanathan Rajan, Naseem Khayum, Syed Suraya, Thota S S Bhaskara Rao, Hari Prasadarao Pydi

**Affiliations:** 1 Department of Research and Development, Robert Bosch India, Bengaluru, 560030 Karnataka India; 2 Department of Mechanical Engineering, Dayananda Sagar University, Devarakaggalahalli, Harohalli, Kanakapura Road, Ramanagara Dt., Bengaluru, 562 112 India; 3 Department of Electrical Engineering, College of Engineering, King Khalid University, Abha, Saudi Arabia; 4 Department of Mechanical Engineering, Madanapalle Institute of Technology & Science (MITS), Deemed to be University, Madanapalle, Andhra Pradesh India; 5 Department of Mechanical Engineering, Bule Hora University, Bulehora, Ethiopia

**Keywords:** Charge air cooler, Engine dynamometer, Transient testing, Calibration correlation, Air pulsation control, Energy science and technology, Engineering

## Abstract

This study presents an experimental investigation aimed at addressing charge air cooler (CAC) delta temperature variation and air pulsation issues encountered during final vehicle calibration on engine dynamometers. Conventional calibration setups typically employ customized water-cooled CAC systems, which often fail to replicate actual vehicle conditions, leading to discrepancies in pressure, temperature, and air–fuel ratio (AFR). To overcome this limitation, a novel air-to-air (A2A) CAC configuration incorporating a dynamically controlled airflow system using a variable frequency drive (VFD) and a closed-loop PID controller was implemented on the engine dynamometer. The proposed setup enables accurate emulation of vehicle charge air temperature and pressure conditions under both stationary and transient operating modes. Experimental results demonstrate a significant reduction in pressure drop from 10 kPa to 1.2 kPa and improved temperature control from 49 °C to 39 °C, resulting in an efficiency improvement of approximately 6–8% compared to conventional water-cooled systems. The optimized CAC performance contributes to improved air density, stable AFR, reduced air pulsation (pumping loss), and enhanced overall engine efficiency. The findings confirm that the proposed methodology effectively replicates real vehicle conditions on the dynamometer, thereby minimizing iterative calibration efforts, reducing development time and cost, and improving reliability in OEM calibration processes.

## Introduction

Automobile vehicles have become essential part in our daily life globally for transporting goods from manufacturer to users of any kind^1^. In view, reduction of CO_2_ or greenhouse gas emission challenge for 21st century is prominently attempted^2^. Various types of modern IC engines available which uses different fuels such as Diesel, Gasoline, CNG, LPG, Hybrid, Biofuels, Hydrogen, Electric and alternative fuels for sustainability^3^. The growing dependence on fossil fuels has led to escalated expenses due to reduced energy security and a substantial increase in harmful greenhouse gas emissions^4^. In recent years^5^, Turbocharger application of IC engines has increased^6^ and has become an inevitable part of the modern high-performance engine due to their usefulness in increasing mass flow and density of air supply to the combustion. This benefits in terms of BSFC, better cycle output^7^, overall efficiency, and reduction in harmful gas emissions^8^ to deliver performance by way of waste heat recovery from exhaust and EGR gases^9^. Combination of Boyle’s law, Charles’ law and Avogadro’s law, generally termed as Ideal Gas Law PV = mRT^10^ states that, as the pressure of air flow increases as well as temperature, CAC or intercooler is essential for engine performance and efficiency. The fresh intake air gets compressed to high pressure thereby harmful warming before air enters engine, density of air decreases which results in lower air mass flow. To overcome this inconvenience and to increase thermal and volumetric efficiency^11^, this air temperature needs to be cooled to optimized level for densification using heat exchanger called intercooler or CAC or after cooler.

**Fig. 1:**
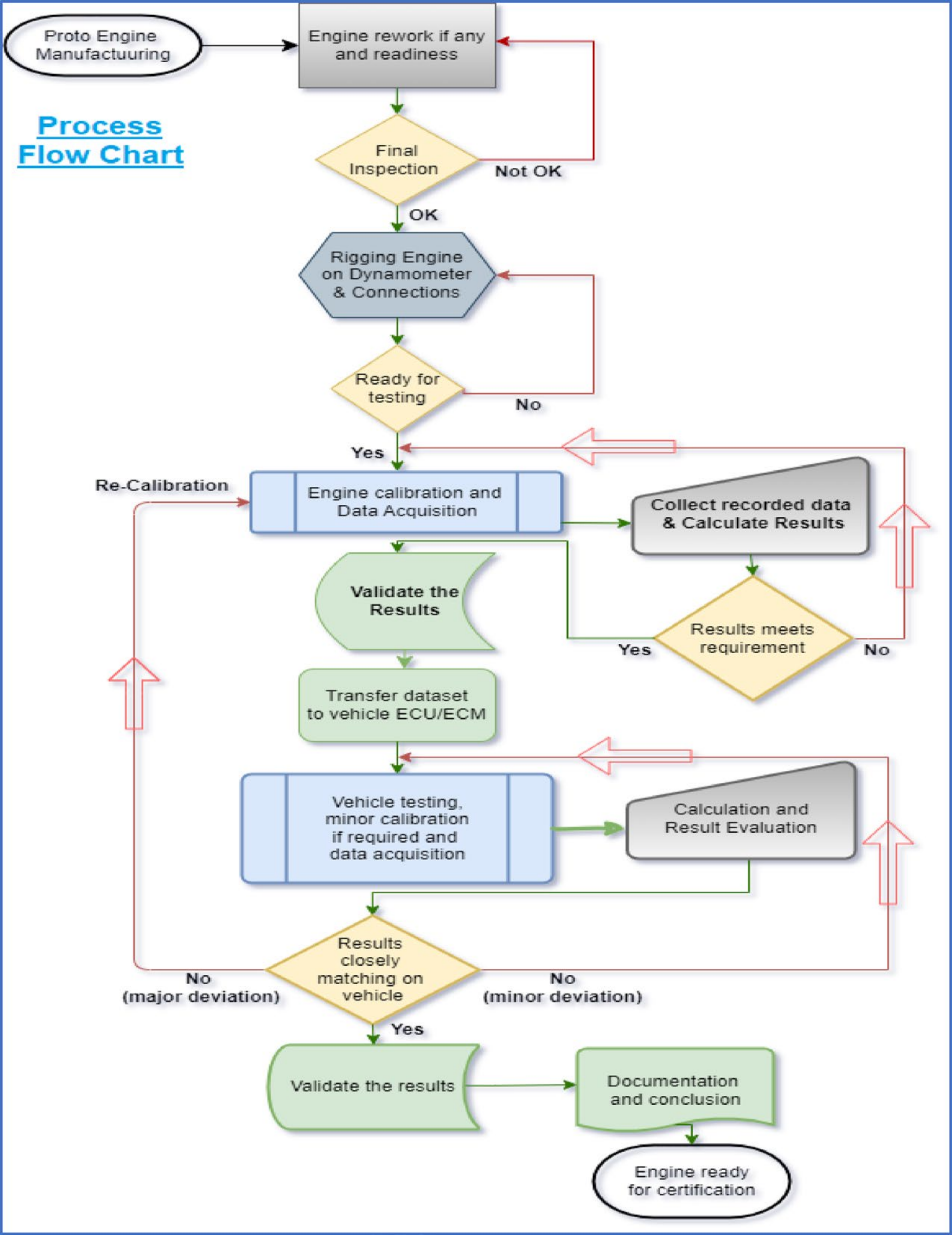
Process flow chart.

Subsequent to engine manufacturing, engine assembly, the process follow through a series of sequential operations (Fig. 1) utilizes engine dynamometers for prototype development, vehicle testing, validation, homologation and, certification which culminates in the release of the vehicle.

OEMs follow a systematic approach to ensure safety standards and performance benchmarks set by a regulatory authority. The combustion in an IC engine is intricate process, relying on intake air mass flow, optimized CAC temperature and air-fuel (A/F) ratio^12^ which is the ratio of air-mass to fuel-mass induced into the IC engines .

**Fig. 2:**
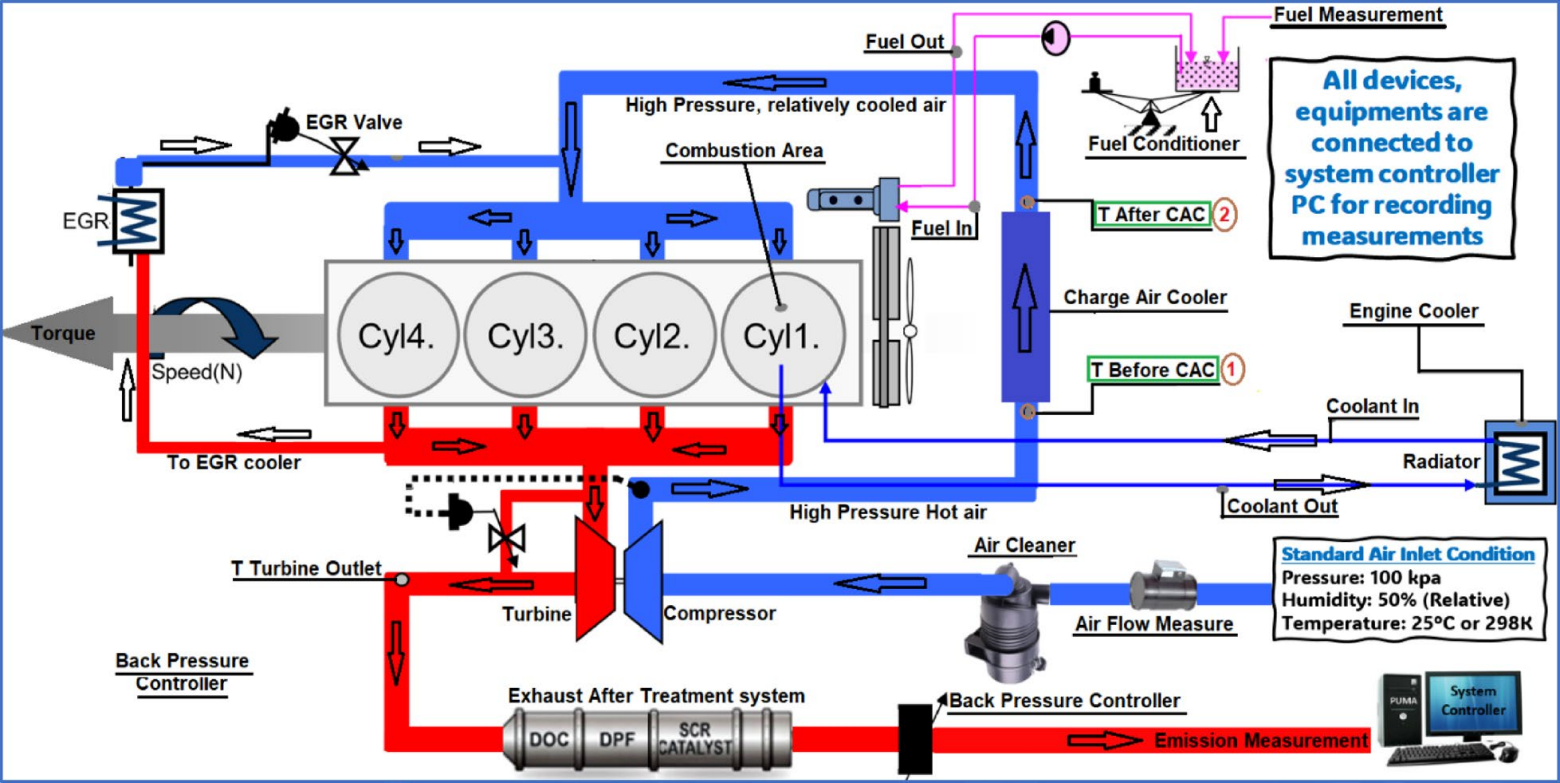
Measurement readiness Schematic.

Figure 2 shows the engine dynamometer setup with sensor placement for measuring key parameters such as intake pressure, temperature, fuel flow, engine speed, and exhaust emissions. The data are acquired and processed for real-time monitoring during stationary and transient tests.

Appropriate measuring equipments like emission bench analyzers to measure exhaust gas constituents such as NOx, THC, CO, CO_2_, smoke meter for FSN, opacimeter for opacity, MSS for soot, PM, PN, and NOx slip NH_3_. Effect of atmospheric pressure and temperature plays a vital role in performance^13^, accordingly The tests were performed^14^ as per STD conditions prescribed (Table 1) and the test ranges for which the correction factors are valid.

**Table 1:** Reference Conditions.

Parameters	Descriptions	Tolerance	Unit
Engine Inlet Air Pressure	100	± 3	kpa
Engine Inlet Air Humidity	50	± 2	%
Engine Inlet Air Temperature	25	± 2	°C
Engine Fuel Inlet Temperature	38	± 2	°C
Max CAC outlet Temperature	48	± 2	°C
Testbed Ambient Temperature	25	± 3	°C
Testbed Ambient Humidity	35	± 3	%
Testbed Ambient Pressure	91	–	kpa

In engine development, dynamometer-based calibration plays a crucial role in optimizing performance, emissions, and fuel efficiency. However, discrepancies between dynamometer conditions and actual vehicle operation, particularly in charge air temperature and pressure, can lead to inaccuracies in calibration and increased development effort. Therefore, there is a need for experimental approaches that can closely replicate real vehicle conditions within controlled test environments.

### Background and motivation

Previous researchers have studied engine intercoolers, CAC, compact heat exchangers, and coolers extensively used in modern turbocharged IC engines. In recent years, different charge air cooling strategies have picked up the pace to cater to the high performance modern turbocharged engines to mitigate environmental pollution norms, improve efficiency, reduce fuel consumption, avoid pre-ignition, high-density air intake management, and detonation on SI engines, during the literature survey for CAC outlet temperature controlling strategy reported by many researchers, most concentrated on CAC outlet temperature management using different methods.

Meanwhile, a 2D model of an air-to-air, cross-flow heat exchanger with finned passage was developed by^15^ by subdividing core into two segments for charge and cooling air. This model was verified for steady-state experiment and then tried on transient operations neglecting the thermal inertia of CAC. When accelerating engine from idle to rated speed at constant load, charge air warming is observed whereas during deceleration from rated to idle results in decreasing temperature trend. A cyclone intercooler performance is better than finned tube air cooler in performance depending on conic angle and inlet pressure as the cooling area is almost 45% more compared to conventional intercooler^16^. A study was conducted^17^ investigated knock mitigation using a water-cooled CAC over an engine speed range of 2000–5000 min⁻¹ (in 500 min⁻¹ increments) and intake pressures of 60–100 kPa. The results showed that increasing engine speed leads to higher charge air temperature and pressure, affecting intake air density. The study concluded that water-cooled CAC systems offer higher cooling effectiveness compared to air-to-air systems. This works better in stationery measurements but no transient measure data available. Similarly in another research, CAC work area divided into internal and external flow regions for characteristic performance analysis of fins^18^, , guide vanes^19^, misaligned fins^20^ using finite element method to establish optimization of CAC exit temperature. Summarizes in this experiment inlet (440 K) and outlet (407 K) regions reaches highest temperatures. In contrast minimum temperature could not reach around the required temperature and measures need to be taken to reduce the target values. According to a researcher^21^, the intercooler system is essential in automotive turbocharged IC engines as it is one of the critical devices in the cooling process. The importance of the CAC cooler is to supply air with optimized temperature to the intake manifold to cater to sufficient mass flow for better combustion with densified air enriched with oxygen content. They use water as a medium for cooling high-temperature inlet charge air to increase a vehicle’s performance and fuel economy. The research explains the limitation of this experiment to theoretical studies due to the unavailability of sufficient technical support for implementing water-cooled CAC on vehicles.

A Simulation was also developed to study the effect of turbocharger and intercooler temperature management utilizing exhaust heat recovery method was also developed^22,23^ using FORTRAN language. Results show that, by increased compressed air pressure and reduced intercooler temperature, the engine power is significantly improved, BSFC reduced from 132 g/kWh to 126 g/kWh and thermal efficiency raised 44% to 47%. However, the CAC outlet temperature increases, power output drops and fuel demand increases^24^ to achieve the same performance.

A researcher^25^ presented an experiment focused on both stationary and transient operating points with an integrated intercooler intake system design for compactness and performance of turbo charged engine Computational fluid dynamics (CFD) modelling to evaluate flow characteristics and heat exchange performance. Simulation and comparison are performed on critical factors such as flow resistance, pressure fluctuations, and temperature distribution. Results indicate that a design featuring central air intake with a baffle exhibits optimal performance. Experimental validation confirms these findings, demonstrating a 5.9 g/kW·h reduction in brake specific fuel consumption at rated conditions and a 20.4% decrease in response time during medium-speed loading when using the integrated system. Due to the stringent regulation in accordance with policy recommendations for G7 and BRICS countries to reduce CO_2_ emissions^26^ along with fuel and emission improvement generally in commercial transport segment. Arif et al.^27^ conducted an experiment on refrigerated intercooler design based on refrigeration cycle as a secondary cooling to further decreases charge air temperature resulting in increased horsepower a performance gain of 15% to 20% was achieved and in another experiment conducted on vehicle using the traditional approach built an autonomous water spray system consists of additional setup^28^, works with 12 V battery operated to run water pump, 3nos sprinkler spray and arduino uno microcontroller for close loop system behavior included with automated switch on and off provision. This semi-automatic close loop system takes input from the sensor placed at the CAC outlet pipe. Based on increase or decrease of CAC outlet temperature the water spray pump activates or deactivates. In addition to this, driver had the choice to activate or deactivate the water sprinkler spray pump manual on/off switch. In continuation researcher experiments using two different liquid media ethyl glycol and hyaluronic acid for cooling charge air using similar setup close looped with the temperature sensor. Experiment conducted with both cooling media one after the other at 20%, 40%, and 60% dilution, results show ethyl glycol and hyaluronic acid when 20% diluted exhibits peak results with 32.38% more efficient^29^. An optimized design of intercooler by introducing twisted-elliptic-tube intercooler instead of segmental baffle plate intercooler which result in higher cooling efficiency with 13.2% increase. Overall heat transfer coefficient increase by 87.8% and pressure drop decrease by 36.9%, the average intercooler exit temperature achieved 7.3 °C lower^30^.

Related to air pulsation or pumping loss, leads to loss of power, performance, torque irregularity and increase in specific fuel consumption and overall efficiency of engine. The pressure loss increases the turbine work to maintain the same boost pressure at intake valves consumes which increases fuel consumption^31^. Another researcher^32^ explained change in pulsation occurs due to improper airflow path by using customized water cooled CAC and piping instead of using vehicle setup. Pulsation occurs when piston moves back and forth it pushes the air into crank case with their underside. This unfavorable CAC layout contributes to concentration of engine lubricating oil mixing with intake air reducing lubricity leads to high temperature at higher operating points. An experiment conducted^33^ comparison between turbine inlet and intercooler piping layout, similarly modified piping layout for intake airflow impacts pressure losses thereby pumping losses during combustion. In comparison water cooled CAC does not match with vehicle CAC due to below reasons,

i. Different volume due to difference in size.
ii. different airflow dynamics due to non-standard piping layout.
iii. High Pressure drop due to difference in sizes of inlet and outlet pipes.

In an experiment by^34^ researcher explains the essentiality for optimizing charge air temperature of turbo charged or high-power supercharged engine. The study based on CFD model and the performance data measured using steady state flow. Experiment used numerical simulation and found heat transfer coefficient can be increased by up to 57%. Main focus was on the study of correlation between air mass flow rate and transverse spacing of tube bundles and analyze heat dissipation. Significant relationship was achieved with correlation values of 0.8464 and 0.8497 respectively using grey correlation theory^35^.

As a result, promoting the adoption of clean and sustainable energy sources is of paramount importance. In this research concentration is on turbo charged Internal combustion engines. To make the engines more reliable^36,37^, turbo chargers were introduced to compresses the fresh air from 200 kPa to 450 kPa depending on the (91.0 kPa to 100 kPa) ambient air condition to increase amount of oxygen for combustion process without increasing the engine size by raising inlet manifold air intake pressure which increases mass flow rate of air for better combustion. Metal air ducts are generally used to transport the hot compressed air from turbo to engine^38^ via intercooler which can withstand this extreme condition also they need to be flexible in design and fit within the narrow space around the engine. The compressed air at high temperature and high pressure means lower oxygen content due to loss of density to overcome this issue intercoolers became integral part of the engine^14^ to make the intake air denser which increases thermal and volumetric efficiency. Unlike super chargers that driven by a drive belt^39^, turbo is driven by the exhaust gas putting it to good use^40^ essentially for performance point of view. In general, vehicle CAC is not present during testing on ED test bench, since it is not established due to many constraints, one of the main reasons is inability to simulate vehicle draft air supply on ED. Few iterations were conducted on PID values to get optimized results. Stationary and transient tests performed with positive results matching with vehicle data. Pressure drop was negligible since the components used and layout maintained was as per vehicle setup.

This research emphasizes on two sections Firstly, “Engine with vehicle CAC delta temperature and pressure optimized on ED to match with vehicle CAC delta temperature and pressure” thereby optimizing AFR of charge air entering combustion chamber. Secondly, minimizing or avoiding air pulsation or pumping effect cause by water cooled customized CAC. Air pulsation refers to unsteady fluctuations in intake air pressure and flow caused by dynamic variations in airflow supply and engine demand, especially under transient conditions. These fluctuations lead to instability in the air–fuel ratio and reduced calibration accuracy on the dynamometer.

### Gaps, novelty, and objectives of the study

Despite the widespread use of engine dynamometers for calibration, conventional setups fail to accurately replicate real vehicle charge air cooler (CAC) temperature and pressure conditions, particularly under transient operating scenarios. This leads to discrepancies in AFR, increased air pulsation, and extended calibration efforts. Existing studies primarily focus on CAC performance optimization or engine testing independently, with limited attention to integrated experimental approaches that emulate real vehicle conditions on dynamometer systems.

To address this gap, the present study proposes an experimental air-to-air CAC system with VFD-controlled airflow and PID-based regulation, enabling dynamic replication of vehicle charge air conditions. The proposed approach significantly improves temperature and pressure control, reduces air pulsation, and enhances calibration efficiency.

**Table 2:** Experimental Metrics.

Parameters	Descriptions	Unit
Engine Speeds	1000–2200 @ 100 steps	rpm
Engine Load	0–680 @ steps of 20	Nm
Coolant Temp	78–82	°C
Engine Oil Temp	90–110	°C
Coolant pressure	40–60	kpa
Fuel Flow rate	11–32	kg/hr
Air flow rate	216–856	kg/hr

The metrics (Table 2) recordings captured during the testing phase to derive a detailed performance analysis and optimization. Subsequently, the data validation against regulatory criteria, and engine calibration is executed utilizing dedicated INCA interface software installed on the testbed PCs compatible with the engine (ECU) Electronic Control Unit. Tests on engine performed by combination of manual and automatic techniques for stationary and transient cycles until performance and compliance targets are met. Details explained in the flow chart (Fig. 1).

When significant deviations from the expected results are detected during vehicle testing, the dataset is again recalibrated on the engine dynamometer for necessary corrective measures and re-evaluated on the vehicle for confirmation. Calibration on the vehicle is fine-tuned, and the results are ensured per legislation guidelines. Certification authorities then evaluate vehicles for performance, overall efficiency, and emissions. After successful validation, OEMs receive COP certification for vehicle release. This intricate process ensures that engines meet safety standards, performance expectations, and regulatory requirements, assuring consumers of reliable and compliant vehicles. It can be observed that while running a turbo engine for a long duration, the temperature of the intercooler increases gradually; either hot charge air enters the intake or the charge air temperature decreases drastically; in both cases, it will reduce the engine’s performance. Keeping the intercooler temperature optimal is essential for the best engine performance.

Objective:

The specific objectives of this study are to:

(i) reduce the deviation in charge air temperature (ΔT) between dynamometer and vehicle conditions,
(ii) minimize intake pressure drop (ΔP) across the charge air cooler system,
(iii) reduce air pulsation and improve air–fuel ratio (AFR) stability,
(iv) decrease calibration rework and testing iterations, and.
(v) evaluate system performance under both stationary and transient operating conditions.

## Methodology

The experimental setup consists of a turbocharged internal combustion engine coupled with an engine dynamometer. The charge air cooler (CAC) is positioned downstream of the turbocharger compressor and upstream of the intake manifold. The airflow path follows the sequence: ambient air → compressor → CAC → intake manifold → combustion chamber.

Key variables measured upstream and downstream of the CAC include intake air temperature, intake air pressure, and airflow characteristics, enabling evaluation of temperature reduction (ΔT) and pressure drop (ΔP) across the CAC system.

### Testing setup and control strategy

In order to evaluate engine according to manufacturer specification for meeting regulatory guidelines, air entering to the intake manifold to be optimized according to the engine speed and load conditions. The manufacturer intercooler not being present on a test bench due to constraint related to fitment and cooling arrangement^41^, water to air heat exchanger of different types are used. Air temperature is maintained using PI global control strategy. An engine test bench is a sophisticated and automated system designed to assess the performance, efficiency, and durability of internal combustion engines^42^. This highly specialized equipment minimizes human intervention during test cycles by utilizing advanced technology such as sensors, data acquisition systems, and control units for precise monitoring and control of key parameters. The feedback variable is the CAC outlet temperature measured using a calibrated sensor, with a sampling rate of approximately 1 Hz for real-time control. The VFD regulates the cooling fan speed, thereby controlling airflow through the CAC. A PID controller is used to maintain the target temperature based on a lookup table, with gains tuned empirically to achieve stable response and minimal overshoot^43^.

### Automation and efficiency

Through automation, engine test benches can conduct extensive tests efficiently and accurately without constant human oversight, thus increasing testing reliability and reducing the risk of errors or inconsistencies in results. By limiting human intervention, these automated systems ensure consistent testing conditions and enable engineers to gather reliable data for analysis and optimization of engine performance, leading to the development of higher quality, cost-effective, more reliable engines for various applications in automotive industries. This article delves into the significance of charge air ΔT in engine dynamometer testing, the simulation of vehicle charge air handling dynamics, and factors influence.

i. Charge air delta temperature.
ii. strategies for enhancing overall efficiency.
iii. The overall impact on engine performance.

### Charge air cooling system design

**Fig. 3:**
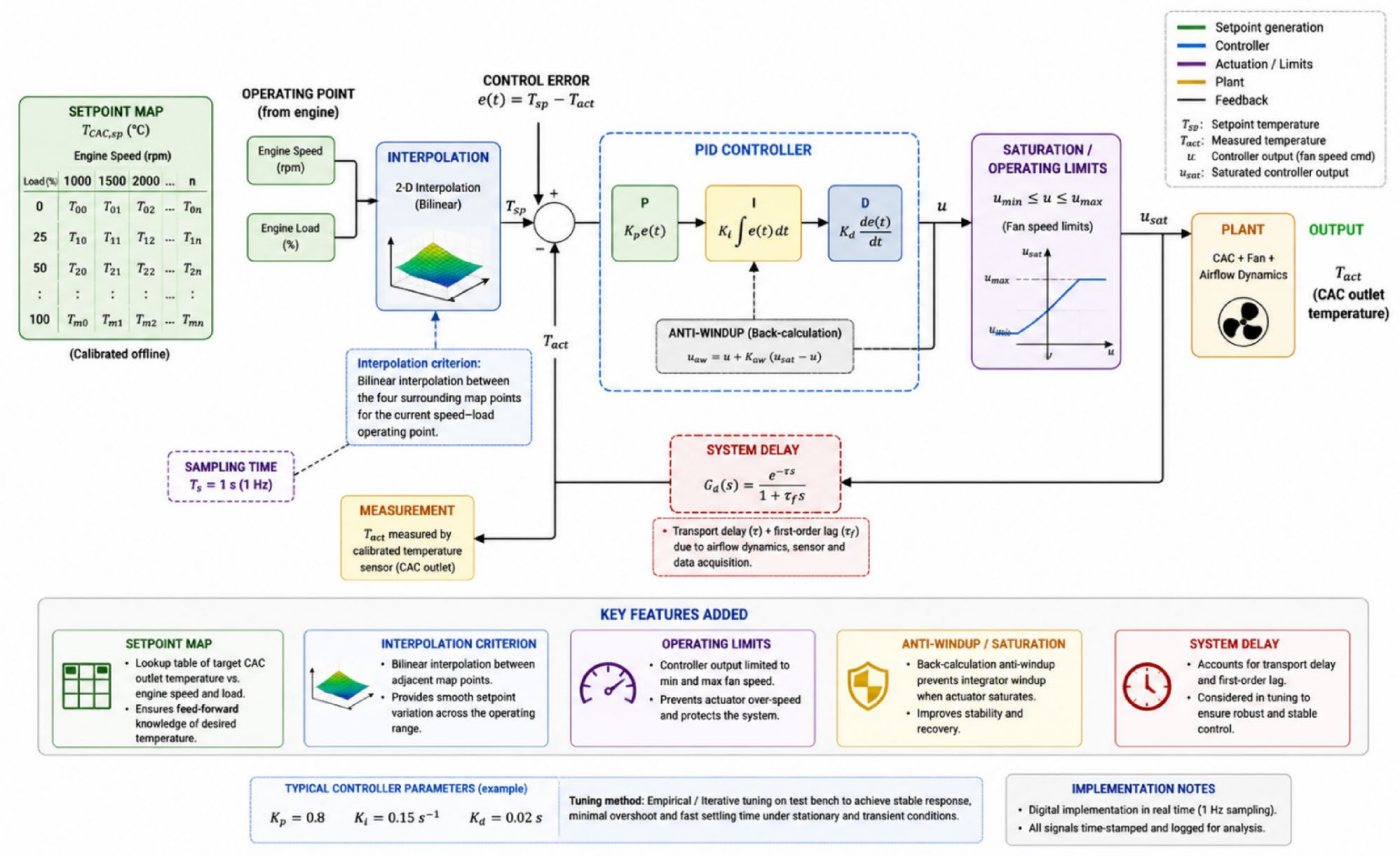
PID Controller.

When it comes to maximizing charge air ΔT efficiency, tuning the charge air cooling systems is key. By ensuring the ideal temperature, pressure, and air flow into the engine maximizes engine efficiency and power. Innovative dynamic modeling for effective charge air handling is designed for controlling system behavior and identifying potential improvements. This involves adopting new strategies and fine-tuning the cooling mechanisms to ensure that the air entering into the engine is at the optimal temperature for combustion. Integrating advanced simulation tools comprising new strategy on engine dynamometer testing enables a comprehensive successful evaluation of vehicle charge air dynamics. Researchers in past have not touched upon these criteria of simulation. In this research Look up table consists of Speed on X axis, Load on Y axis and CAC demand outlet temperature on Z axis, close-looped PID controller (Fig. 3) has been used to control the VFD motor to change the rotational speed according to the difference based on demand and actual CAC outlet temperature.

**Fig. 4:**
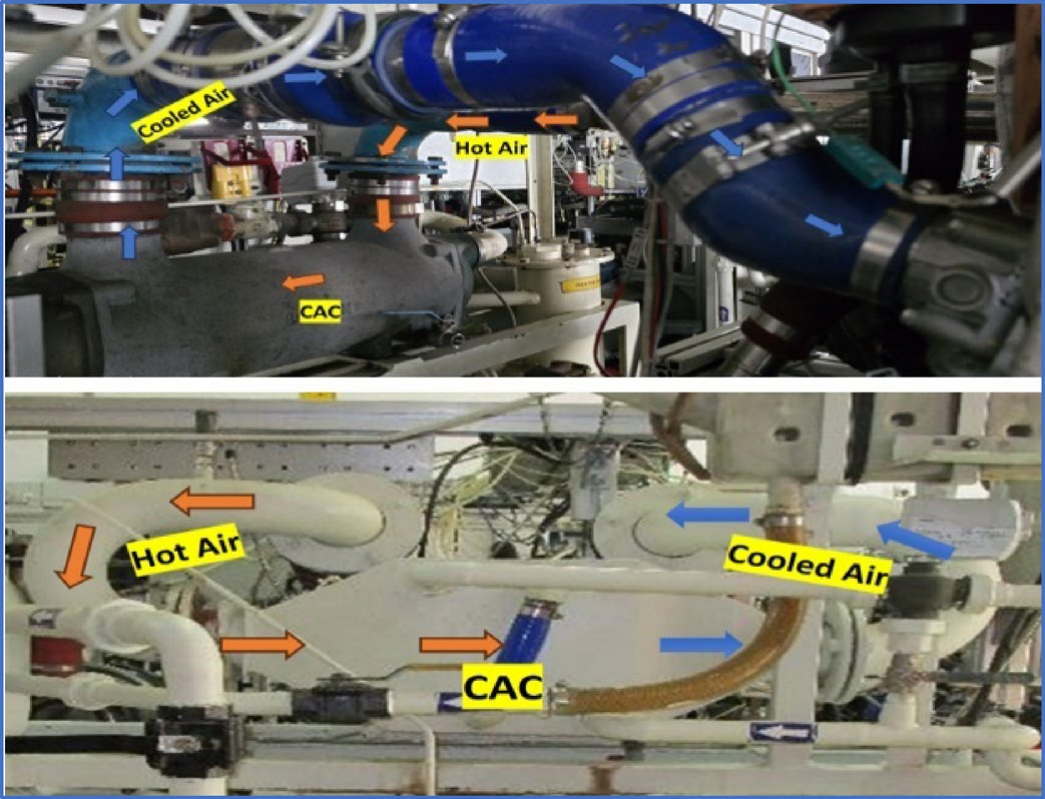
Customized intercooler.

By exploring these key aspects, the aim of this research is to focus on charge air temperature reduction and related cooling control strategies for testing engine on dynamometer with specially designed aerodynamic charge air cooling arrangement. This strategy avoids repeated calibration for pulsation correction of intake air flow due to change in charge air flow by using vehicle CAC instead of customized intercooler as in Fig. 4.

### Control and feedback mechanisms

**Fig. 5:**
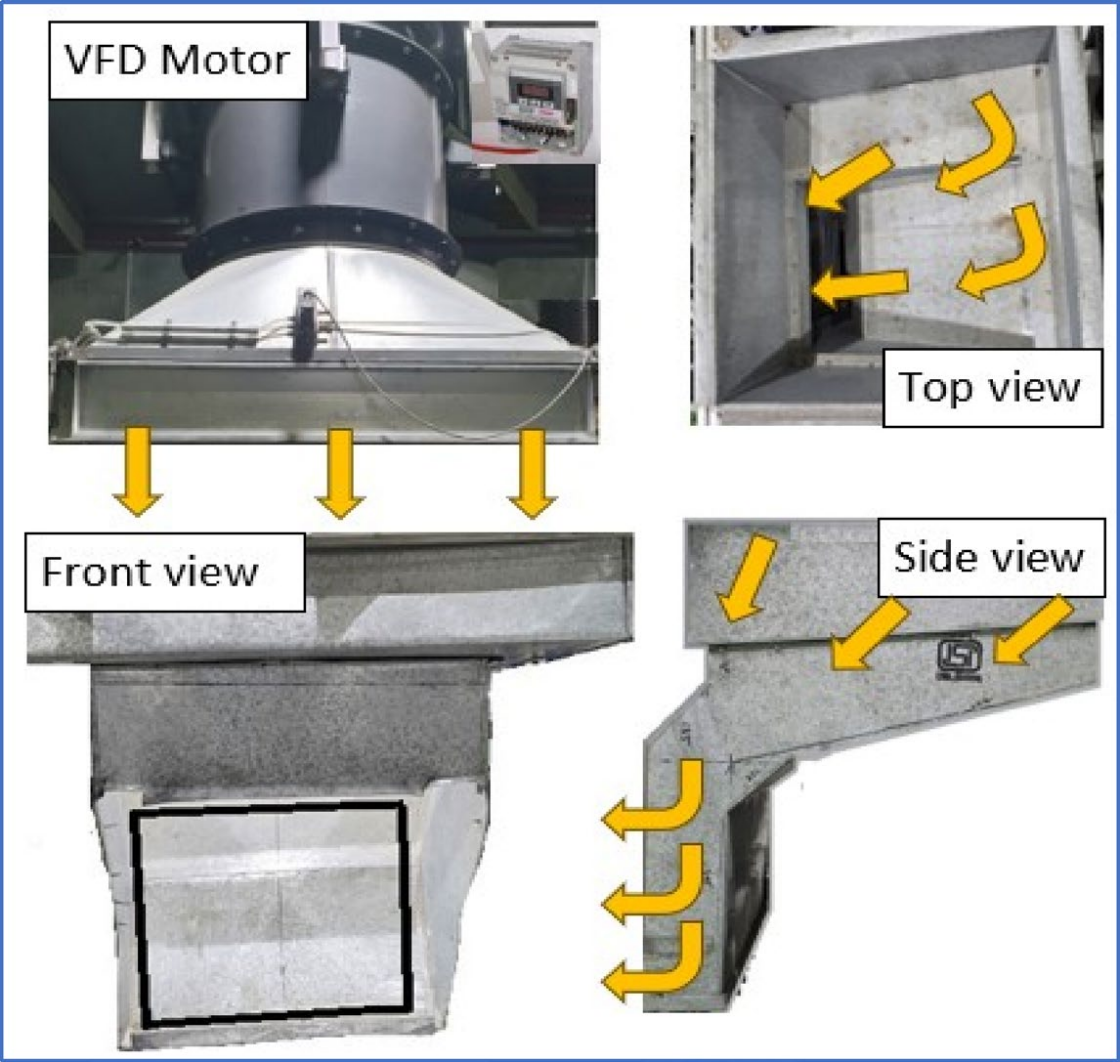
Air to Air Cooling Duct setup

**Fig. 6:**
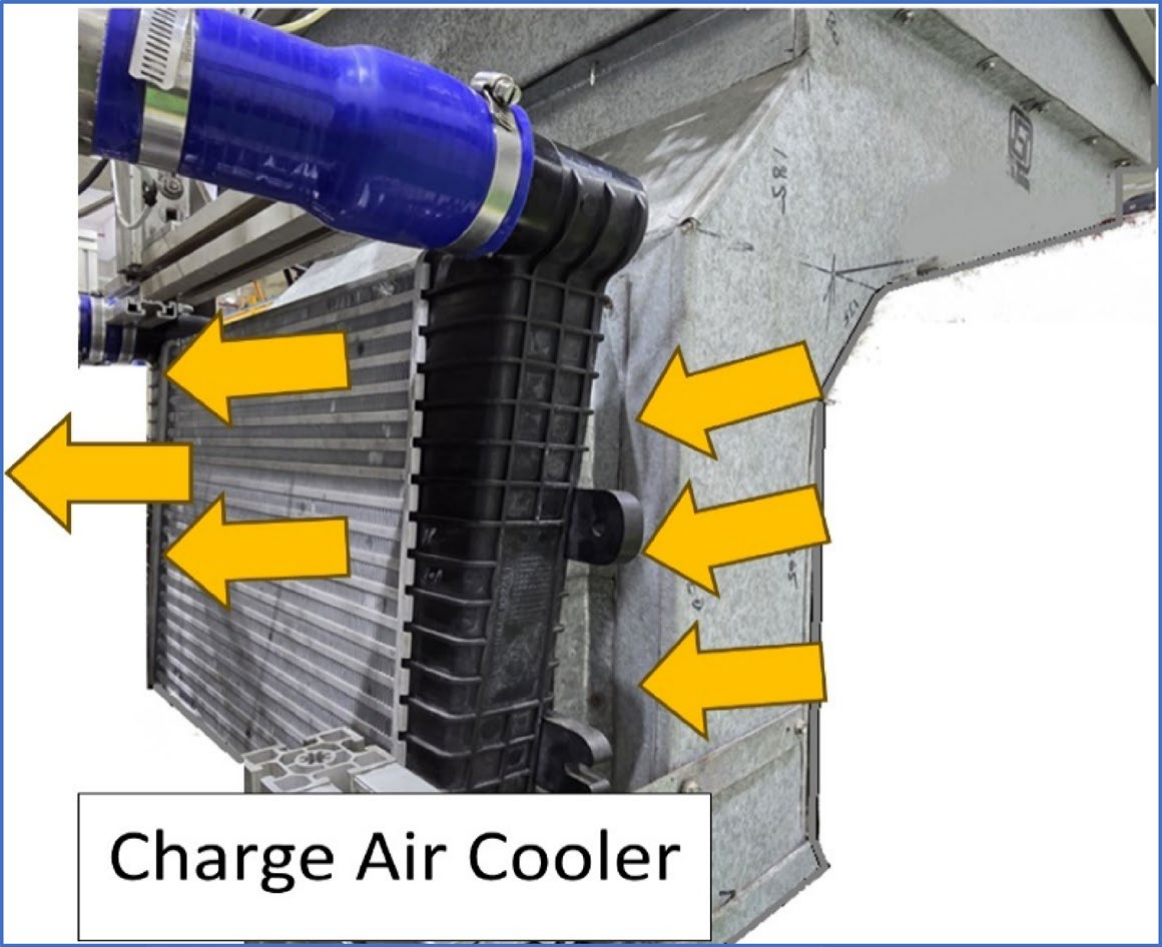
Vehicle CAC setup.

Controlling a system can be summarized as controlling one or several values within the system. The system uses a closed-loop control strategy. In this research, a setup (Figs. 5 and 6) consists of a PID Controller is used for controlling VFD drive control with fan blades based on integrated demand input data fed in engine testing automation software in form of lookup table. The PID control architecture was enhanced with acceleration feedback through the close-loop^42^ feedback mechanism with an integral control method designed to maintain a dynamic demand output value by continuously adjusting an input signal based on the difference between the actual and target values.

This difference, known as the error signal, drives the controller’s three main components: proportional (P), integral (I), derivative (D).

Intercooler is fixed according to the vehicle layout with vehicle parts only to get the best simulation results aerodynamically designed duct is fabricated on engine pallet which can connect to, blower docking arrangement and intercooler assembly ensuring air escape passage is bare minimal.

**Fig. 7:**
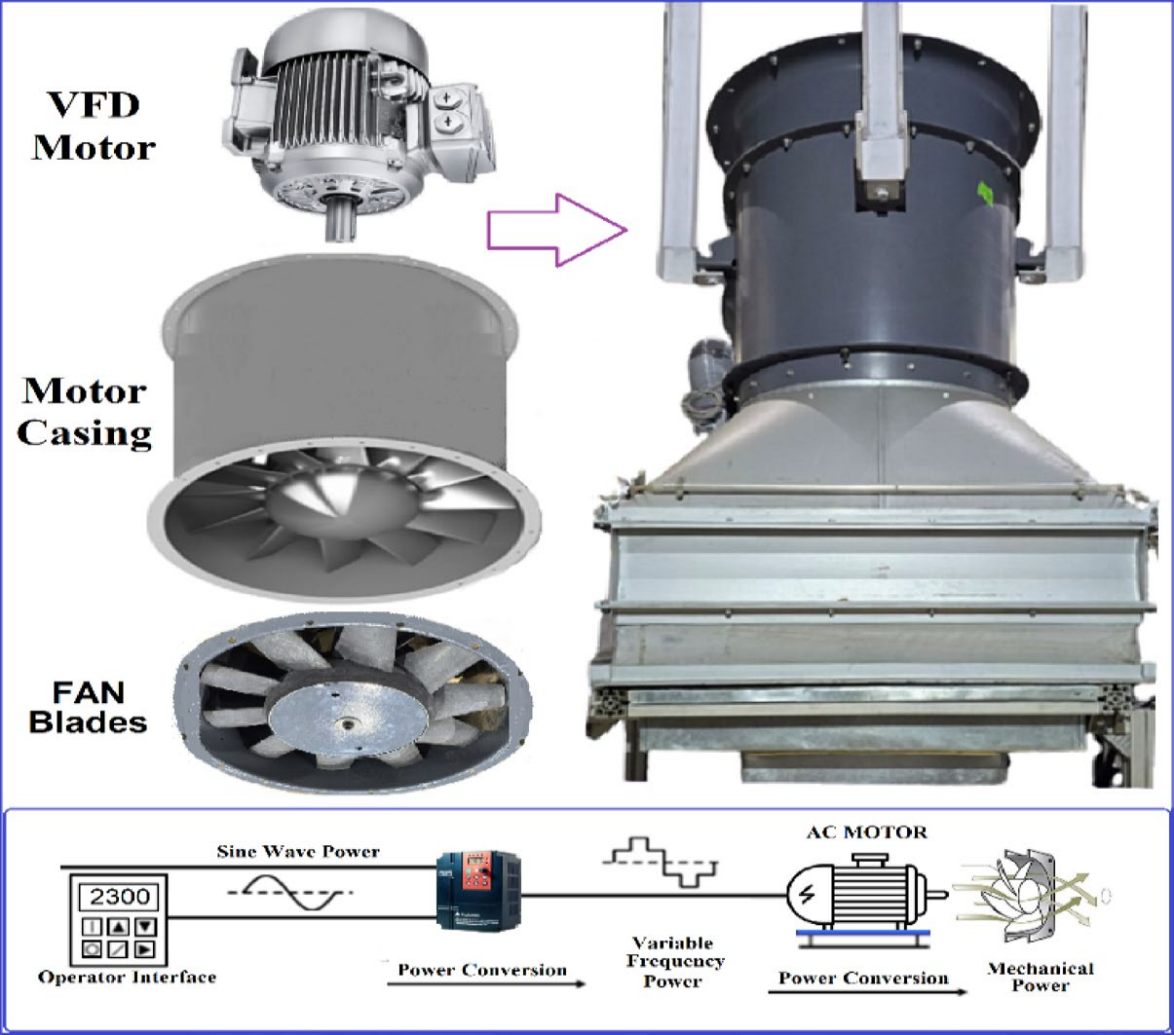
VFD drive setup.

CAC dynamic cooling airflow arrangement (Fig. 7):

- VFD (Variable Frequency Drive) Motor fan fitment done at rotary end.
- Sheet metal duct prepared (specially designed layout) for aerodynamic flow of cooling air.
- Fitment of brackets for Proper alignment of duct and intercooler.
- Look up Table generated and integrated into testbed control software.
- Interconnections and PID controller configuration completed and validated.
- Verified the setup for its correct functionality as per the requirement.

**Fig. 8:**
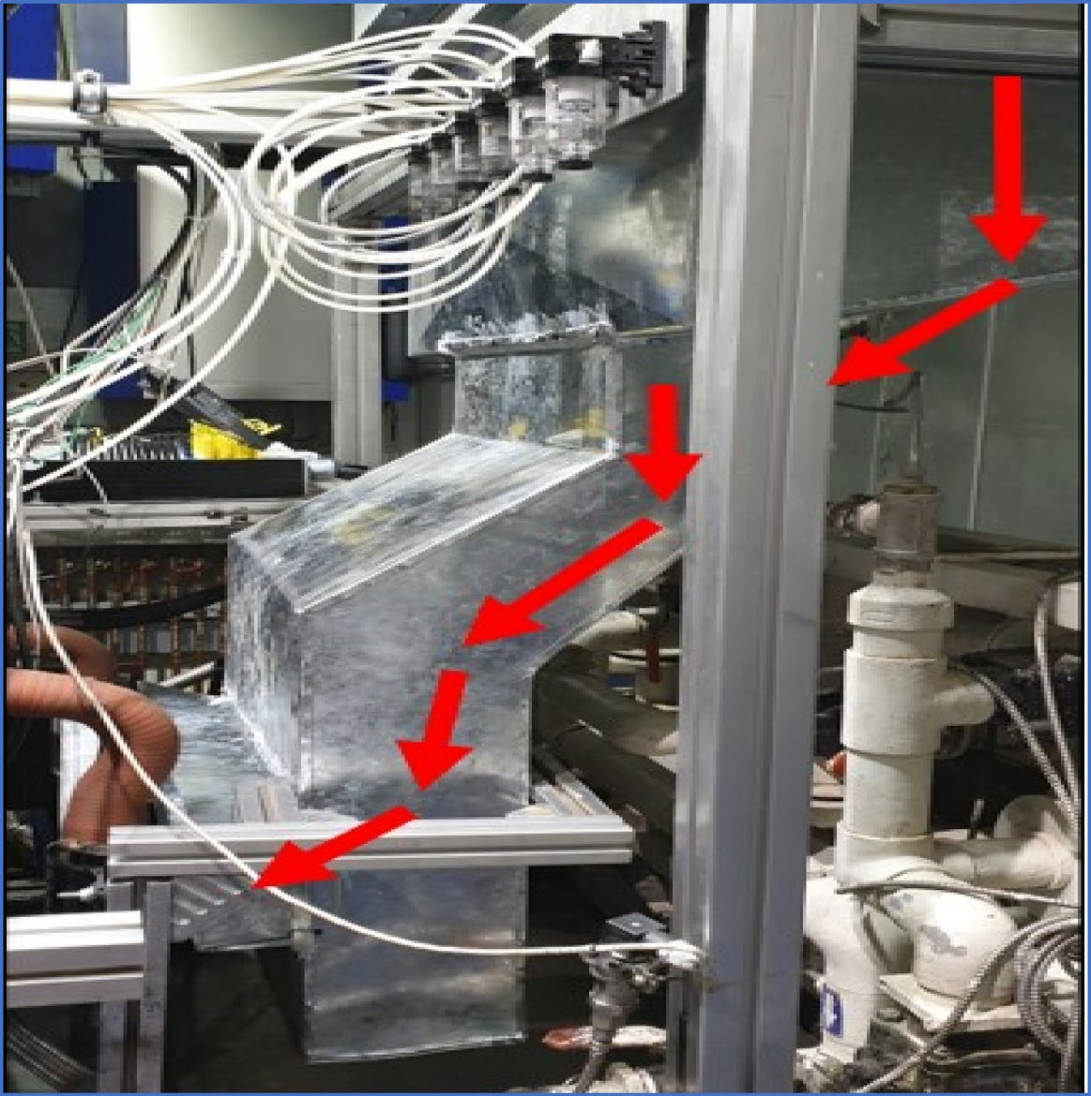
CAC Cooling Air.

Flow Path:

Figure 8 shows the VFD-driven airflow system, where a variable frequency drive motor with an integrated fan (10–2300 RPM) is mounted above the air duct to supply controlled airflow to the charge air cooler. The setup is aligned with the engine pallet to ensure unobstructed and efficient cooling airflow.

**Fig. 9:**
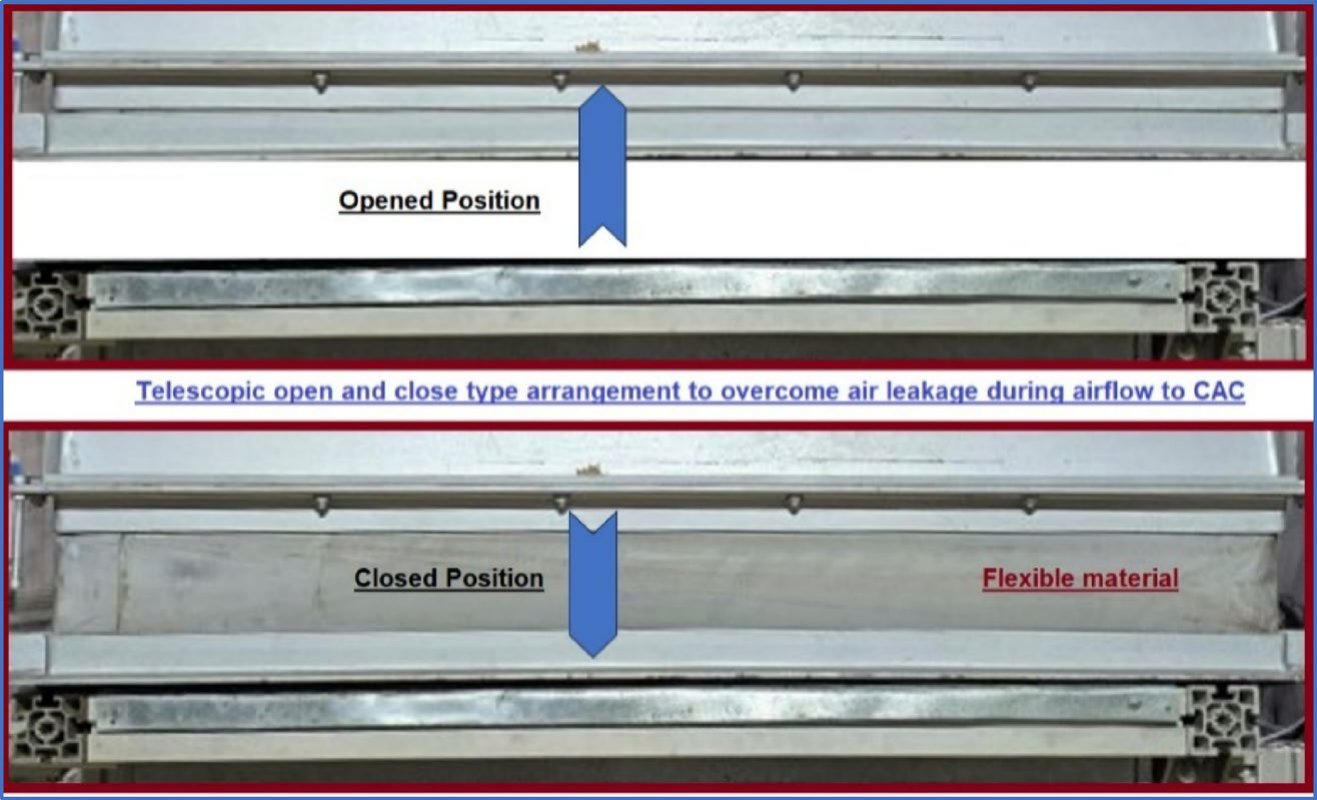
Telescopic open close setup.

Figure 9 shows the telescopic docking arrangement used to connect the VFD-driven airflow system with the air duct inlet. This mechanism ensures proper alignment and minimizes air leakage, enabling efficient and directed airflow to the charge air cooler. This proper alignment of air inlet duct and telescopic arrangement avoids air (escape) leakage and supports for air flow at the designated duct path for cooling Charge Air effectively. The VFD motor is programmed to automatically switch on at a nominal speed when the engine RPM crosses above 600. VFD integrated with PID, operates based on engine speed and simulates the real driving condition of vehicle set up.

According to the look up table, the fan dynamically changes its speed based on demand temperature on **Z** axis picking up operating point interpolating from Engine Speed on **X** axis and Load on **Y** axis, actual temperature of CAC outlet is maintained with a tolerance of ± 1 °C. Engine calibration is performed to meet the legislation requirement with engineering margins and after final Stationary and Transient test is performed, the acquired data is plotted and evaluated in accordance with legislation guidelines.

The engine meets overall performance and requirements on the dyno calibration activity, the calibration dataset is transferred to the vehicle for validation in vehicle-related tests. If substantial deviations are observed in the results, further calibration on the engine dyno is required. Otherwise, calibration fine-tuning is performed on the vehicle to conclude the tests. The vehicle is ready for certification tests at the competent authority establishment.

**Fig. 10:**
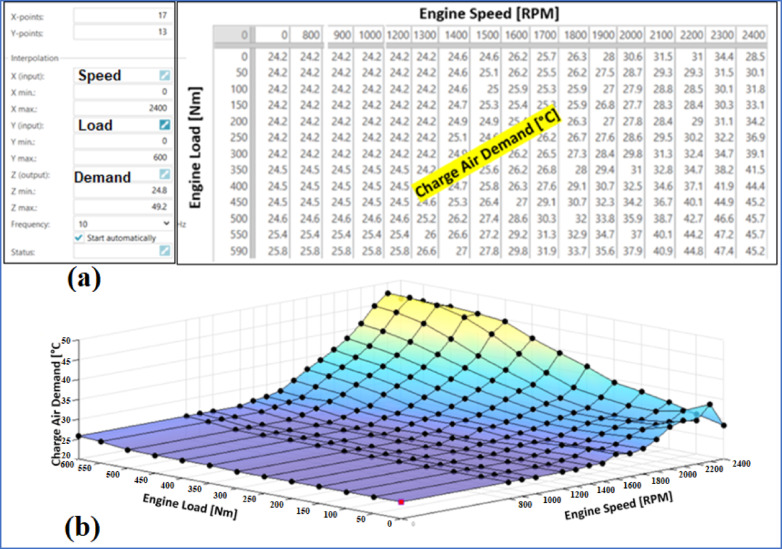
Lookup Table.

Figure 10 illustrates the lookup table integrated into the control system, where engine speed (X-axis) and load (Y-axis) determine the desired CAC outlet temperature (Z-axis). This reference map is used by the PID controller to dynamically regulate the VFD-driven airflow and maintain the target temperature. Engine Speed on **X** axis, Load on **Y** axis, and demand CAC temperature on **Z** axis. Engine calibration is performed to meet the legislation requirement with engineering margins there after final Stationary and Transient test is performed, the acquired data is plotted and evaluated in accordance with legislation guidelines. The engine meets overall performance and requirements on the dyno calibration activity, the calibration dataset is transferred to the vehicle for validation in vehicle-related tests. If substantial deviations are observed in the results, further calibration on the engine dyno is required. Otherwise, calibration fine-tuning is performed on the vehicle to conclude the tests. The vehicle is ready for certification tests at the competent authority establishment.

## Results & discussions

The operating points were selected based on representative engine speed and load conditions covering both stationary and transient regimes. For each condition, experiments were repeated multiple times to ensure repeatability, with a stabilization period maintained before data acquisition. The collected data were averaged and used for subsequent performance and comparative analysis.

**Table 3:** Engine Specifications.

Parameter	Description	Unit
Maximum Torque	680@ 1200–1900	N-m@rpm
Rated Power	133 @ 2400	kW @ rpm
Engine	CV Heavy Duty	
Engine Idle Speed	800	Rpm
Cooling Type	Water	-
Bore	10.5	cm
Stroke	11.55	cm
No. of Cylinders	6	-
Swept Volume	5998	cc
Connecting Rod Length	21.85	cm
Compression Ratio	11.5	-
Engine Firing Order	1-5-3-6-2-4	-

**Fig. 11:**
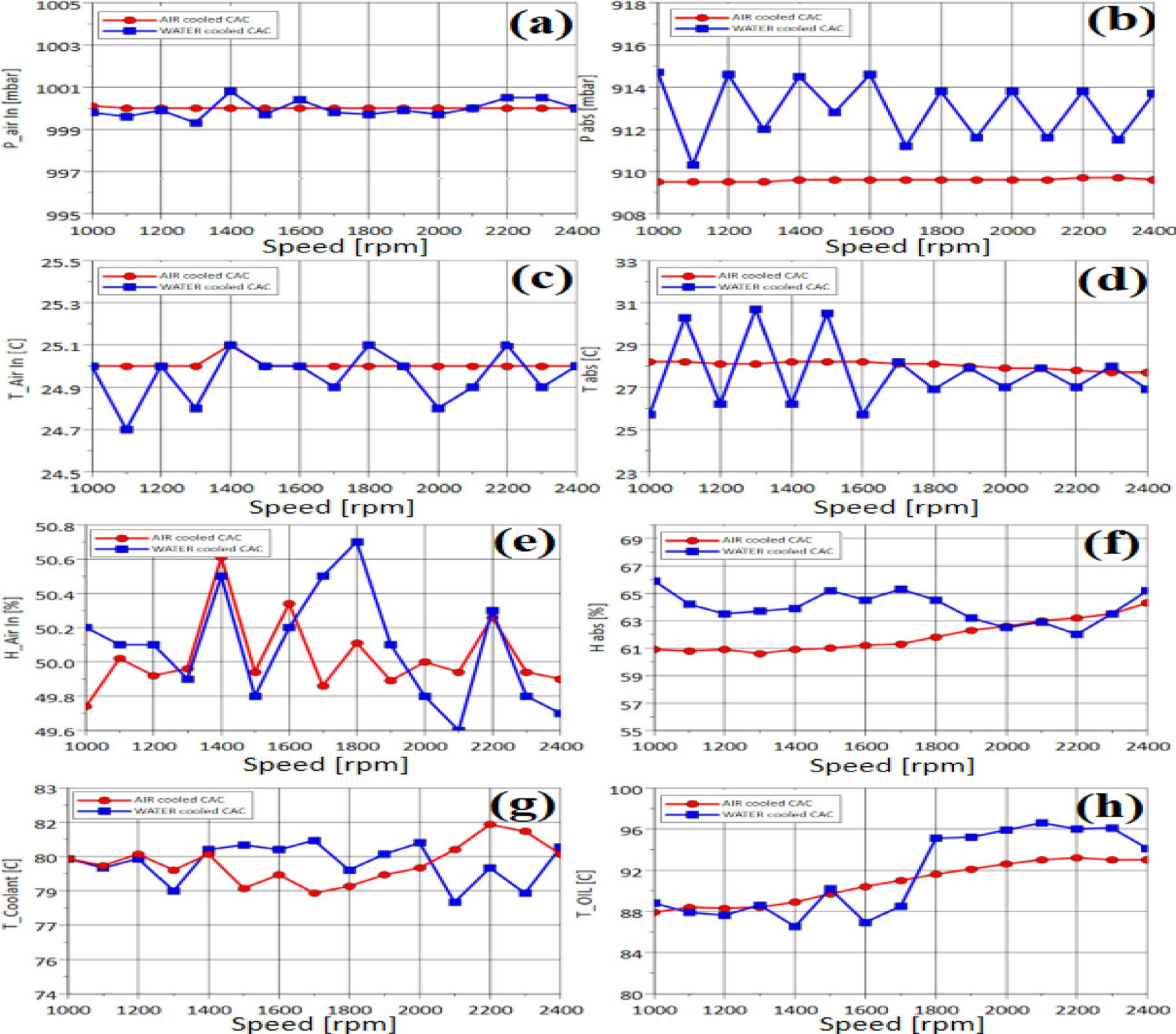
Boundary Condition.

In general, the density depends on temperature and pressure of the charged air. For better correlation between engine and vehicle outlet parameters, standard boundary conditions were maintained (Fig. 11). As soon as the temperature of the charge air increases, density of the air decreases and vice-versa. This plays a vital role in deciding air-fuel ratio which contributes to effective BSFC. Selected engine specification for this research is mentioned in Table 3, and uncertainties of measurements in Table 4 based on equipments are documented as reference for calculations.

**Table 4:** Uncertainties of Measurements.

Parameter	Instrument	Range	Accuracy	Resolution	Uncertainty	Propagation Method
Fuel Flow Rate	Flow Meter	0–10 kg/h	± 1%	0.01 kg/h	± 1.5%	Root sum square (RSS)
Intake CAC temperature	k-type	0–200 °C	± 1.0 °C	0.1 °C	± 1.5%	RSS
Engine Speed	Optical Encoder	0–6000 rpm	± 1 rpm	1 rpm	± 0.2%	RSS
Intake Pressure	Pressure Transducer	0–200 kPa	± 1 kPa	0.1 kPa	± 1.2%	RSS
Airflow Rate	Hot-wire Anemometer / Flow Sensor	0–50 m³/h	± 2%	0.1 m³/h	± 2.5%	RSS
Exhaust Emissions (CO, HC, NOx)	Gas Analyzer	As per spec	± 1–2%	-	± 2%	RSS

### Conversion from engine speed to vehicle speed

The conversion applied in this section is used to relate vehicle speed to the corresponding airflow conditions required on the engine dynamometer. This enables accurate replication of real vehicle operating conditions within the test setup. The converted parameter directly influences the estimation of inlet air temperature and airflow characteristics, which are critical for evaluating CAC performance in terms of temperature reduction (ΔT) and pressure drop (ΔP). This approach ensures that the experimental conditions closely mimic actual vehicle scenarios, thereby improving the reliability of the analysis.

**Table 5:** Data from OEM site.

Parameters	Descriptions	Unit
Gear Ratio	1.5	-
Final Drive Ratio	2.6	-
Wheel Circumference	2.03071	m
Wheel Radius	0.32667	m
4th Gear Ratio	1.5	-
Tire Selected	**220/55/R16**	-

Below data obtained from the OEM and other values are calculated using standard formulas. Used 2 formulas^44,45^ for data conversion Table 5 from engine speed to vehicle speed Tire specification: 220 mm, 55% and 16 inches) calculation

1 $${\text{Side wall height}}\,=\,{\text{H }}={\text{ 22}}0{\text{ mm }} \times {\text{ }}0.{\mathrm{55}}\,=\,{\text{121 mm}}$$

2 $${\text{Rim diameter}}\,=\,{{\mathrm{D}}_{\mathrm{r}}}={\text{ 16}} - {\text{inch }} \times {\text{ 25}}.{\mathrm{4}}\,=\,{\mathrm{4}}0{\mathrm{6}}.{\text{4 mm}}$$

3 $${\text{Wheel radiusrw}}\,=\,0.{\mathrm{32667}}$$

4 $${\text{Wheel diameter}}\;{\mathrm{dw}}={\text{ }}\left( {{\text{2 }} \times {\text{ H}}} \right){\text{ }}+{\text{ }}{{\mathrm{D}}_{\mathrm{r}}}{\mathrm{mm}}$$

5 $${\text{Wheel Circumference}}\;{\mathbf{C}}\,=\,\pi \times {\text{ dw}}$$

Using (1), (2), (3), (4) and (5) Formula:

6 $$\:\mathrm{V}\mathrm{e}\mathrm{h}\mathrm{i}\mathrm{c}\mathrm{l}\mathrm{e}\:\mathrm{S}\mathrm{p}\mathrm{e}\mathrm{e}\mathrm{d}\:(\mathrm{k}\mathrm{m}/\mathrm{h})=\frac{RPM\:\:\times\:\mathrm{W}\mathrm{h}\mathrm{e}\mathrm{e}\mathrm{l}\:\mathrm{C}\mathrm{i}\mathrm{r}\mathrm{c}\mathrm{u}\mathrm{m}\mathrm{f}\mathrm{e}\mathrm{r}\mathrm{e}\mathrm{n}\mathrm{c}\mathrm{e}\times\:\:60}{Gear\:Ratio\:\times\:\:\:\mathrm{F}\mathrm{i}\mathrm{n}\mathrm{a}\mathrm{l}\:\mathrm{D}\mathrm{r}\mathrm{i}\mathrm{v}\mathrm{e}\:\mathrm{R}\mathrm{a}\mathrm{t}\mathrm{i}\mathrm{o}\:\:\times\:\:\:1000}$$

7 $$\:\mathrm{V}\mathrm{e}\mathrm{h}\mathrm{i}\mathrm{c}\mathrm{l}\mathrm{e}\:\mathrm{S}\mathrm{p}\mathrm{e}\mathrm{e}\mathrm{d}\:(\mathrm{k}\mathrm{m}/\mathrm{h})=\frac{RPM\:\times\:{\uppi\:}\times\:\:3.6\:\times\:\:\mathrm{r}\mathrm{w}}{30\:\times\:\:\:\mathrm{F}\mathrm{i}\mathrm{n}\mathrm{a}\mathrm{l}\:\mathrm{D}\mathrm{r}\mathrm{i}\mathrm{v}\mathrm{e}\:\mathrm{R}\mathrm{a}\mathrm{t}\mathrm{i}\mathrm{o}\:\:\times\:\:\:\mathrm{G}\mathrm{e}\mathrm{a}\mathrm{r}\:\mathrm{R}\mathrm{a}\mathrm{t}\mathrm{i}\mathrm{o}}$$

### Charge air temperature

For complete results of stationary temperature measurement, below equation is used^44,46^.

8 $$\:\mathrm{E}\mathrm{f}\mathrm{f}\mathrm{i}\mathrm{c}\mathrm{i}\mathrm{e}\mathrm{n}\mathrm{c}\mathrm{y}\:=\frac{(Inlet\:Temperature\:-\:Outlet\:Temperature)}{(Inlet\:Temperature-Ambient\:Temperature)}\times\:100\:$$

**Fig. 12:**
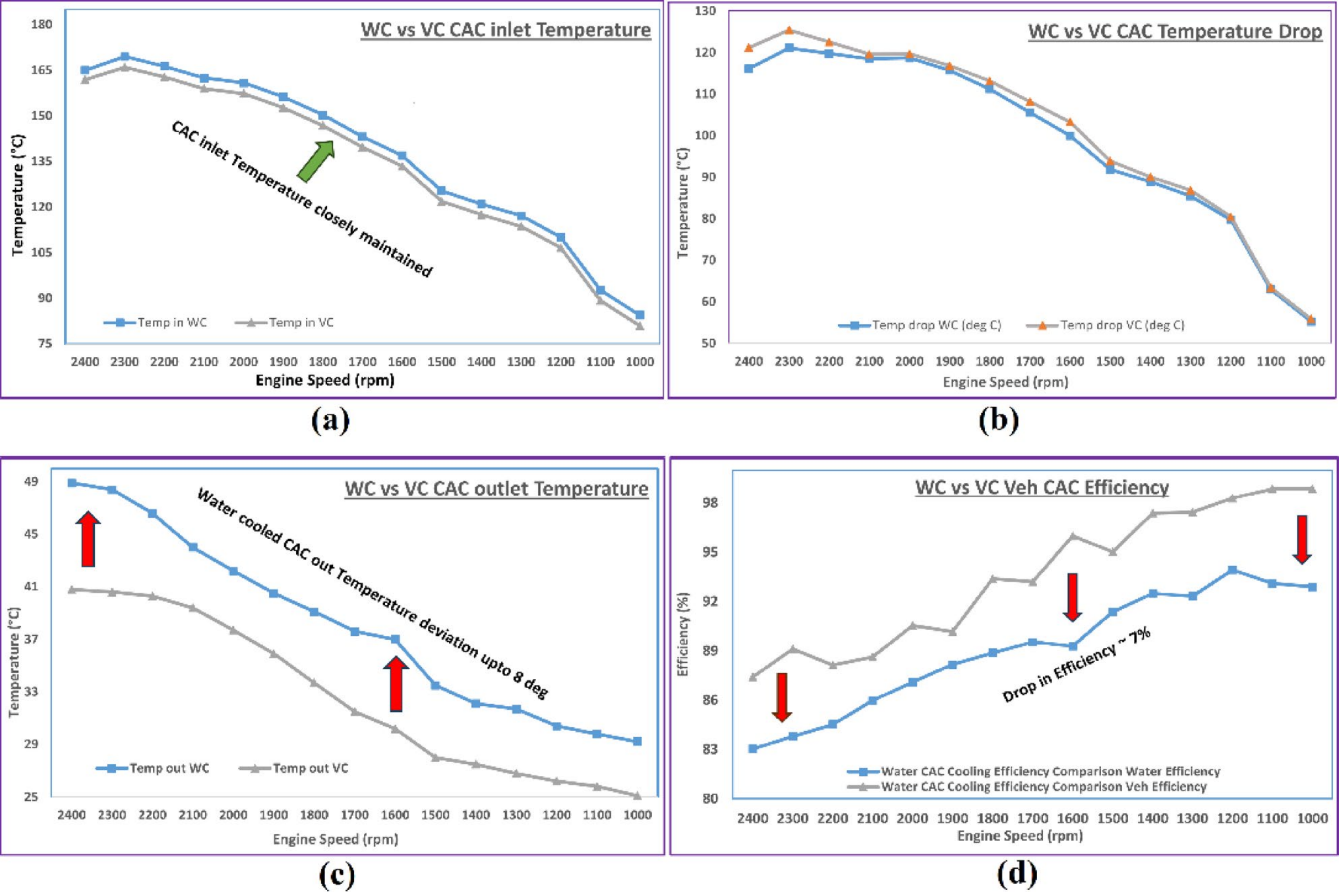
Water Cooled Pressure & Temperature Comparison.

**Fig. 13:**
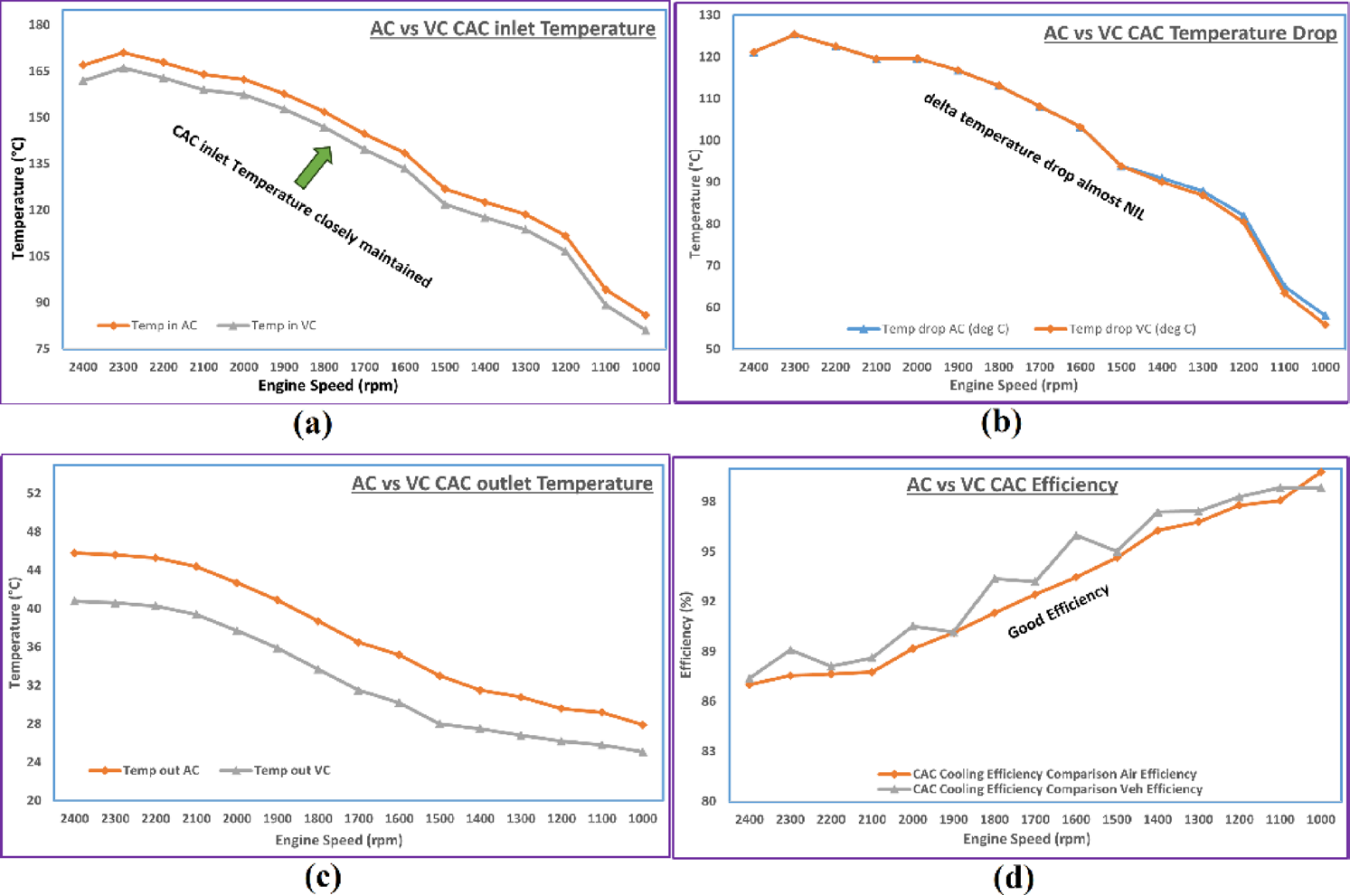
Air Cooled Pressure & Temperature Comparison.

**Fig. 14:**
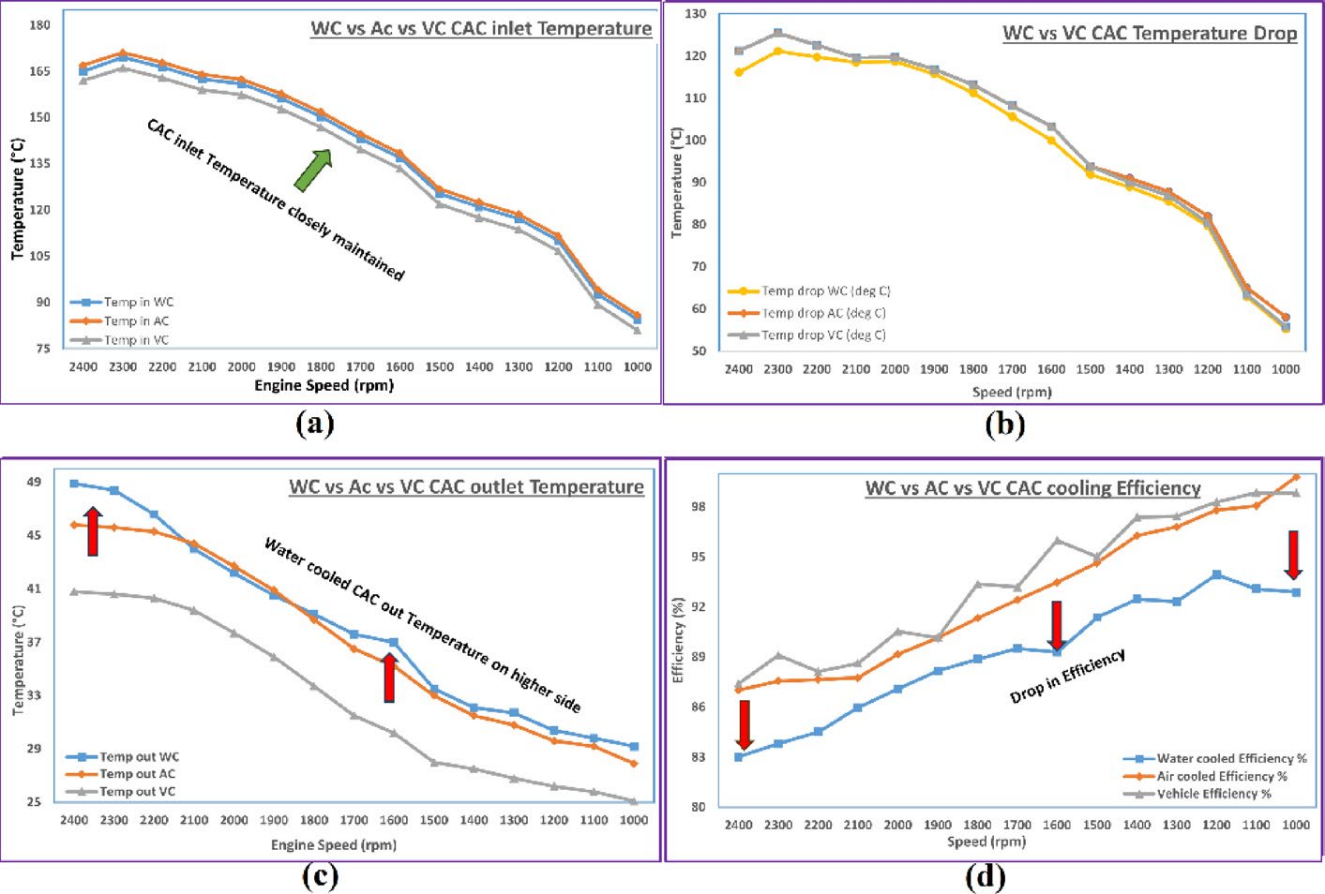
Air Cooled Pressure & Temperature Comparison.

Calculated temperature values of water-cooled CAC (Fig. 12) are distinctly presented as (a) inlet temperature, (b) delta temperature, (c) outlet temperature and (d) efficiency, and temperature values of air-cooled CAC (Fig. 13) are distinctly presented as (a) inlet temperature, (b) delta temperature, (c) outlet temperature and (d) efficiency, similarly temperature values of vehicle CAC (Fig. 14) are presented as (a) inlet temperature, (b) delta temperature, (c) outlet temperature and (d) efficiency.

**Fig. 15:**
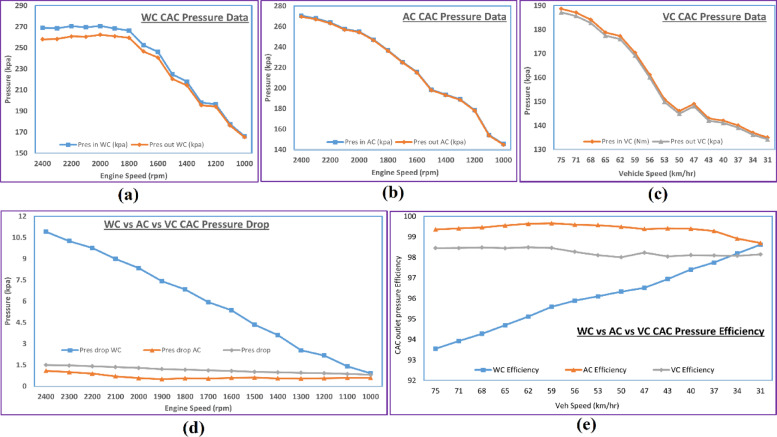
Temperature comparison.

**Fig. 16:**
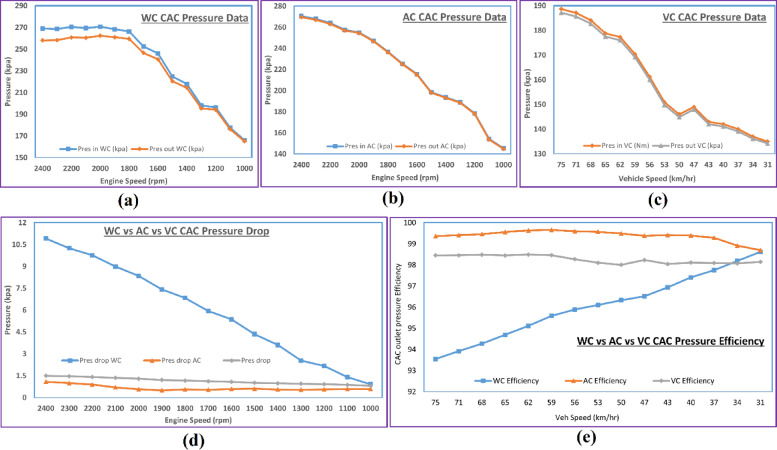
Air Cooled Pressure Drop

The recorded and calculated stationary measure data for the dependent variable, calculation shows the efficiency for comparison with water-cooled, air-cooled and Vehicle data for CAC outlet temperature in Fig. 15.

### Charge air pressure

Similarly, the below equation is used to calculate the CAC pressure efficiency^45,46^

9 $$\:\mathrm{E}\mathrm{f}\mathrm{f}\mathrm{i}\mathrm{c}\mathrm{i}\mathrm{e}\mathrm{n}\mathrm{c}\mathrm{y}\:=1-\:\frac{\left(CAC\:inlet\:Pressure\:-CAC\:Outlet\:Pressure\right)}{\left(CAC\:inlet\:Pressure-Ambient\:Pressure\right)}\times\:100\:$$

**Fig. 17:**
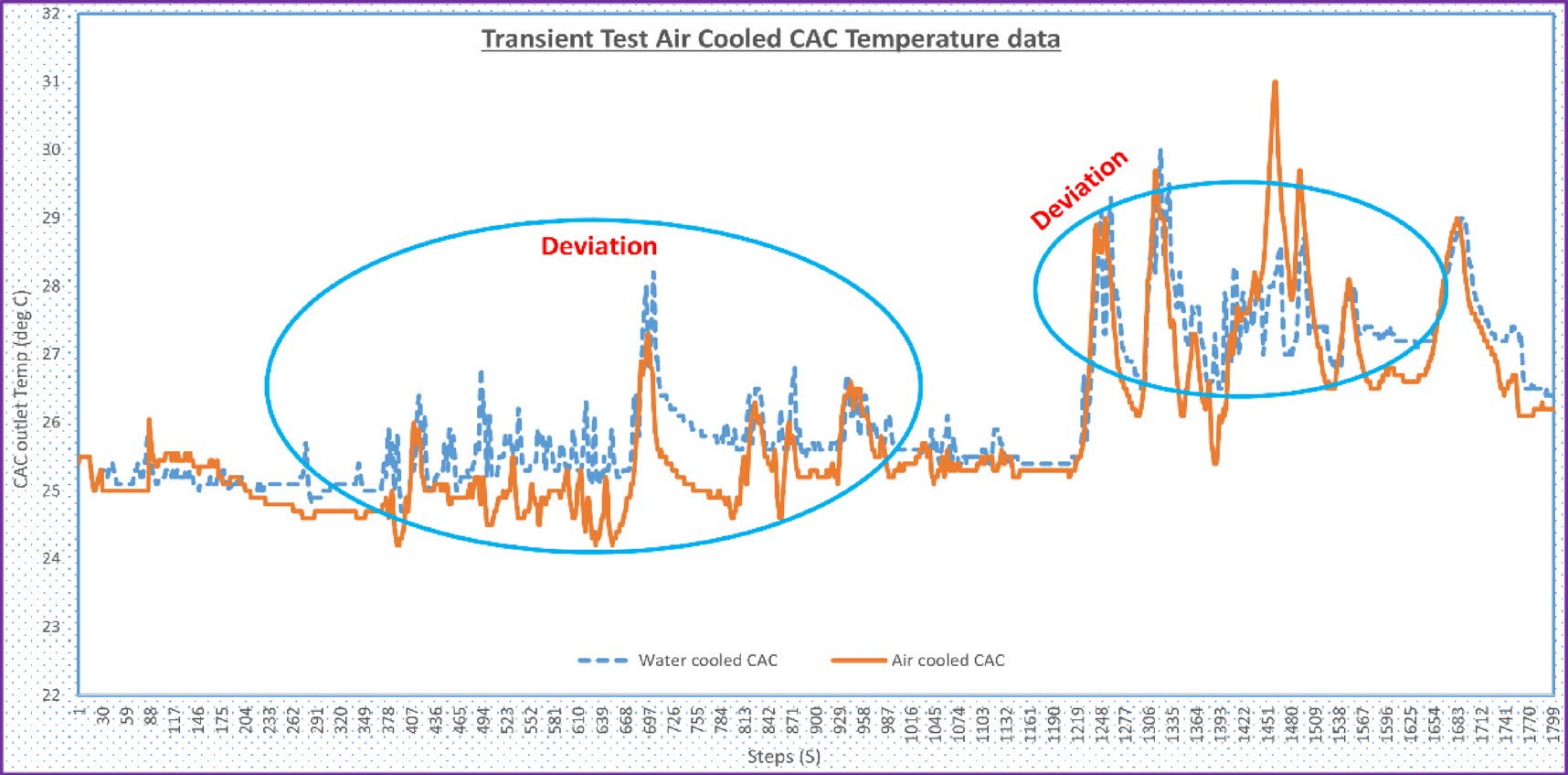
Transient test temperature.

**Fig. 18:**
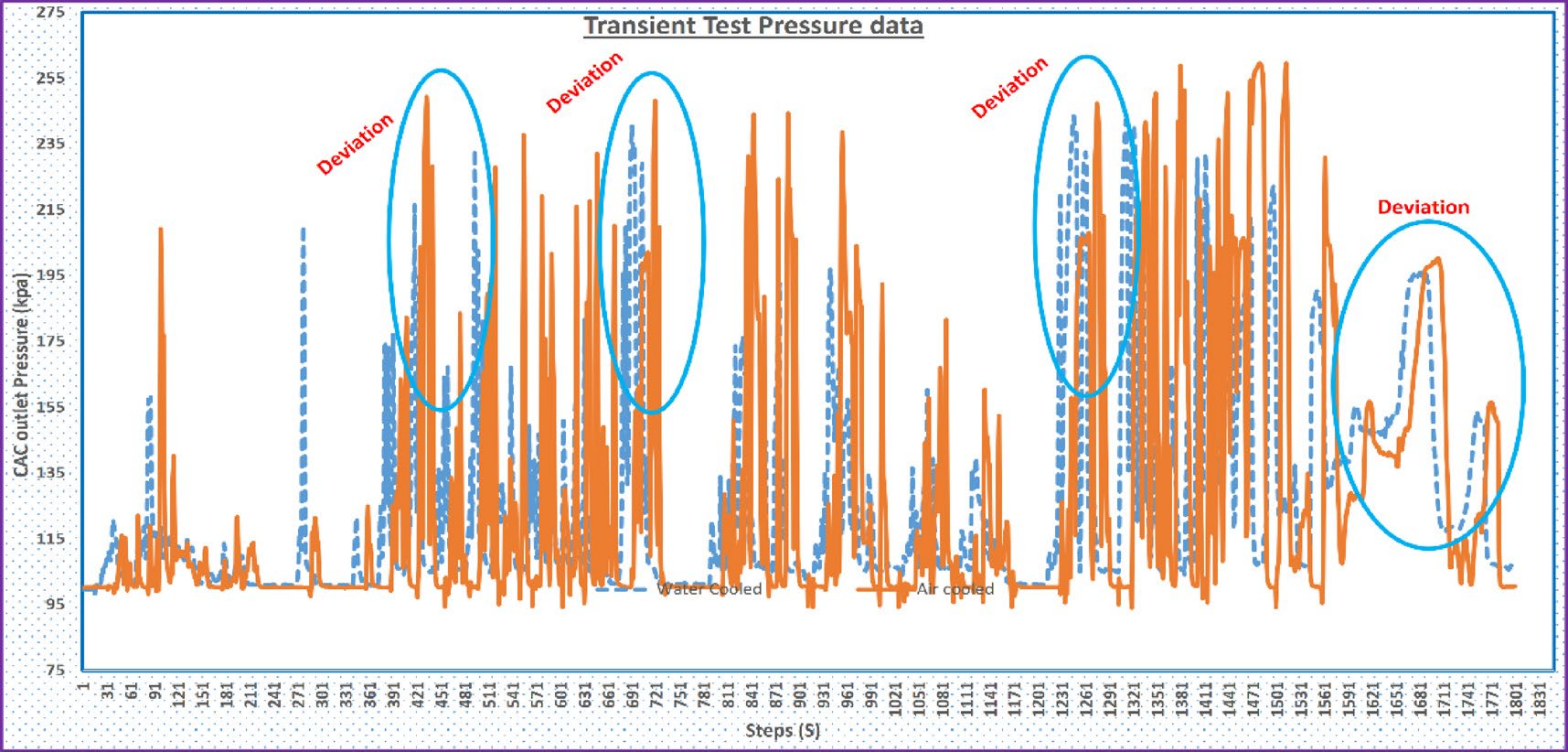
Transient test pressure.

**Fig. 19:**
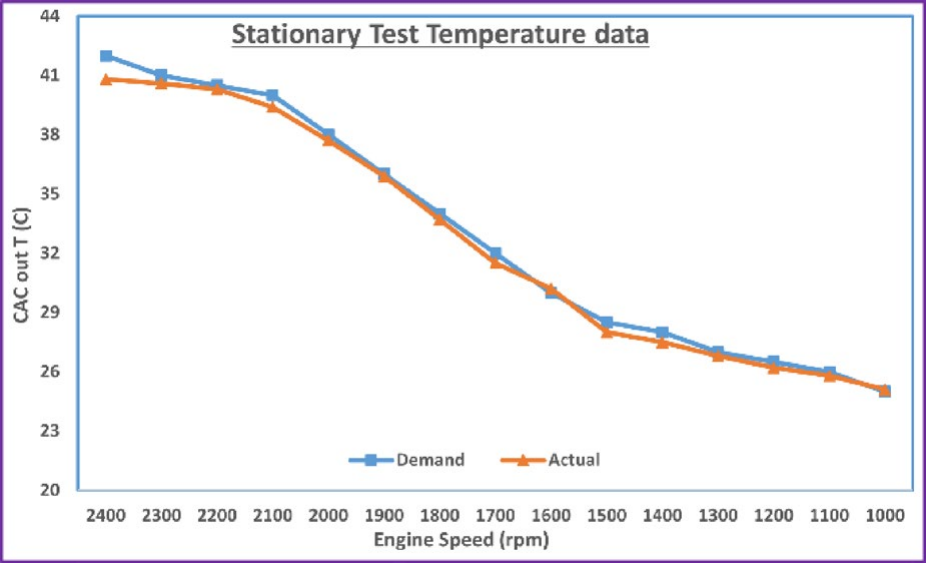
Stationery test temperature.

Note: Standard conditions are adopted during testing for better correlation as per standards^20^, for all calculation purpose, engine speed and load equivalent vehicle speed, pressure difference for engine intake 100 kPa in ED and vehicle intake 91.8 kPa (Bengaluru Altitude 850 m) ambient are also considered as vital parameters.

**Fig. 20:**
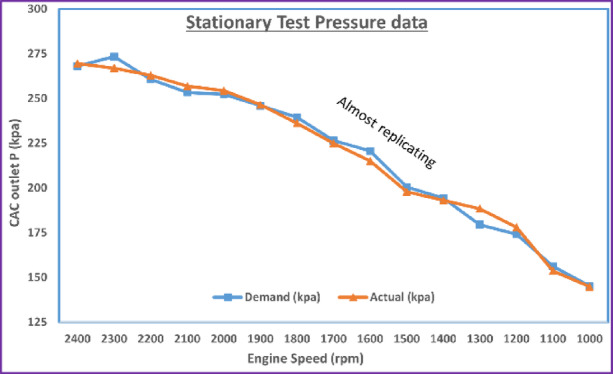
Stationery test pressure

Individual CAC delta pressure values are plotted in Fig. 16 for each variant of CAC. Inlet and outlet pressure are presented as (a) water-cooled, (b) air-cooled, (c) vehicle CAC. Whereas comparison of pressure between all 3 variants are presented as (d) delta and (e) efficiency. During transient operating points temperature (Fig. 17) and Pressure (Fig. 18) behavior shows water cooled CAC under performs in comparison with air-cooled CAC. Further, the stationary test data for temperature and pressure are shown in Figs. 19 and 20 respectively.

The data obtained from the parametric calculations made for the intercooler analyzed by using the computer program. The results of this study are presented in tables and graphs. From the data plotted, it can be observed that while running turbo engine for a long duration or distance, temperature of intercooler will increase, and hot air entered intake, when charge air temperature decreased drastically, in both case it will reduce performance of engine. It is important to keep the temperature of intercooler optimal for the best performance of the engine. With this experiment, results, and analysis, it is found the outcome is significantly efficient and meeting the specific requirement. Improvement in CAC outlet temperature control increased air density contributes to the better combustion, better emission results, overall engine efficiency, significantly matched and results comparable with vehicle calibration results.

The report will be discussed in two main parts. Firstly, the current CAC system with development of a new optimizing strategy, simulations and comparison is explained. Secondly, illustrate the improvement of the system, advantages in vehicle testing area will be discussed where none of the researchers touched upon this area. This research numerically investigated the impact considering the recorded results during tests.

## Summary

In the present paper, innovative A2A duct integrated CAC cooling design established. The heat-transfer performance of the conventional water-cooled shell and tube type CAC output and air-cooled vehicle CAC output are experimentally investigated and compared, both CAC are practical products meets the cooling requirement. Through the analysis, the following conclusions are drawn:

- An application of this innovative air-cooled vehicle charge air cooling design for ED result in considerable benefits in terms of good temperature control, a higher heat-transfer coefficient.
- This setup demonstrated higher cooling capability by an increase of ~ 8% as well as negligible delta pressure which minimizes future calibration process.
- Vehicle CAC provides more realistic output and does not need any pulsation correction as the hardware used is vehicle and replicates when validated on vehicle testing.
- Under the same operating conditions, the overall heat-transfer coefficients enhanced, and the heat-transfer area and air pressure drop are reduced from 10 kPa and 1.2 kPa.
- Water-cooled CAC is less efficient in transient operating points and also requires additional dataset calibration for pulsation (air-system) correction during vehicle calibration validation.
- This research significantly promises contribution towards lower vehicle calibration cost, effort, and time.

Exploring advanced cooling technologies, implementing innovative strategies for more efficiency, leveraging advanced simulation techniques allows engineers to evaluate different scenarios and optimize the charge air control strategy before implementation, integrating smart control systems provides automation and close looping, and refinement of experimental methodologies are potential areas that can lead to more efficient and eco-friendly results to push the boundaries of automotive technology. As we look to the future, there are promising avenues for further research and development related to charge air handling for cleaner combustion, and positive environmental impact in this field with potential to drive innovations that enhance both environmental sustainability and driving experience. Future work may include adding smart structures like piezoelectric for more accurate sensing purposes.

The proposed charge air cooling strategy demonstrates strong industrial applicability, particularly in OEM engine calibration processes, by enabling accurate replication of vehicle charge air conditions on engine dynamometers. This approach significantly reduces calibration time, cost, and iterative testing efforts, thereby improving overall development efficiency. The present study is limited to a specific engine configuration and operating range, and further validation under broader engine platforms and real driving conditions is required. Future work will focus on extending the methodology to transient driving cycles, integrating advanced control strategies, and evaluating its applicability to next-generation powertrain systems.

## Data Availability

The data will be made available upon request to the corresponding authors.

## Variables and parameters

AFR: Air–fuel ratio
BSFC: Brake specific fuel consumption (kg/kWh)
e: Control error (°C)
L: Engine load (%)
u: Control signal
ΔT: Temperature difference across CAC (°C)
ΔP: Pressure difference across CAC (kPa)
t_s: Sample time (s)
T_in: Inlet air temperature (before CAC) (°C)
T_out: Outlet air temperature (after CAC) (°C)
T_sp: Setpoint CAC temperature (°C)
T_act: Actual CAC outlet temperature (°C)
K_i: Integral gain
K-p: Proportional gain
P_in: Inlet air pressure (before CAC) (kPa)
P_out: Outlet air pressure (after CAC) (kPa)
N: Engine speed (rpm)
Q_air: Air flow rate (m³/s)
m_f: Mass flow rate of fuel (g/s)
ρ_air: Air density (kg/m³)
V_air: Velocity of air (m/s)
η: Efficiency (%)

## Abbreviations

A2A: Air-to-Air (cooling system)
CAC: Charge Air Cooler
ED: Engine Dynanometer
ECU: Engine Control Unit
PID: Proportional-Integral-Derivative Controller
OEM: Original Equipment Manufacturer
VFD: Variable Frequency Drive

## Declarations

### Use of generative AI

ChatGPT (GPT-5, OpenAI, 2025) was used to assist in language refinement, grammar correction, and improving the readability of the manuscript. The authors have thoroughly reviewed and verified the content to ensure the accuracy and integrity of the scientific information presented.

## References

[1] Pandey, K. K. et al. Performance enhancement and emission reduction in spark ignition engines using carbon nanotubes and zinc oxide hybrid nanoparticles. *Environ. Prog. Sustain. Energy* **45**(2), e70241 (2026).

[2] Nandakishora, Y., Khayum, N., Patra, C., Christydass, S. & Salau, A. O. Energy, exergy, and environmental analysis of a solar-assisted cryogenic carbon capture process. *J. Renew. Sustain. Energy* **18**(2), 026301 (2026).

[3] Khayum, N. et al. Influence of carbon free gaseous ammonia induction on combustion, performance and emissions in an agricultural diesel engine operated on dual fuel mode. *Sci. Rep.* **16**, 2572 (2025). 41390702 10.1038/s41598-025-32413-z PMC12820111

[4] Jacob, S. et al. Performance and emission analysis of a diesel engine fueled with cashew nut shell-derived biodiesel and its blends. *Eng. Proc.* **114**(1), 16 (2025).

[5] Panting, J., Pullen, K. R. & Martinez-Botas, R. F. Turbocharger motor-generator for improvement of transient performance in an internal combustion engine. *Proc. Inst. Mech. Eng. D J. Automob. Eng.* **215**, 369–383. 10.1243/0954407011525700 (2001).

[6] Khayum, N., Shaik, J. H. & Nandakishora, Y. Machine learning and deep learning prediction of in-cylinder pressure and heat release rate in an NH3–fueled diesel engine. *Appl. Therm. Eng.* **17**, 128684 (2025).

[7] Khayum, N., Anbarasu, S. & Murugan, S. Optimizing waste heat recovery in a dual-fuel diesel engine through thermoelectric generation and heat pipe integration. *J. Renew. Sustain. Energy* **16**(6), 063103 (2024).

[8] Khayum, N., Anbarasu, S. & Murugan, S. A role of the combined effect of fuel injection parameters on a dual fuel diesel engine. *Mater. Today Proc.* **1**(47), 2726–2736 (2021).

[9] Lion, S., Michos, C. N., Vlaskos, I. & Taccani, R. A thermodynamic feasibility study of an Organic Rankine Cycle (ORC) for heavy-duty diesel engine waste heat recovery in off-highway applications. *Int. J. Energy Environ. Eng.* **8**, 81–98. 10.1007/s40095-017-0234-8 (2017).

[10] Garrett, S. L. Ideal Gas Laws. In *Understanding Acoustics: An Experimentalist’s View of Sound and Vibration* 333–356 (Springer, Cham, 2020). 10.1007/978-3-030-44787-8_7.

[11] Uzun, A. A parametric study for specific fuel consumption of an intercooled diesel engine using a neural network. *Fuel* **93**, 189–199. 10.1016/j.fuel.2011.11.004 (2012).

[12] Kamarudin, R. et al. Influence of oxyhydrogen gas retrofit into two-stroke engine on emissions and exhaust gas temperature variations. *Heliyon* **10**, e26597. 10.1016/j.heliyon.2024.e26597 (2024). 38434285 10.1016/j.heliyon.2024.e26597 PMC10907674

[13] Koh, W. C., Mazlan, N. M., Rajendran, P. & Ismail, M. A. A computational study to investigate the effect of altitude on deteriorated engine performance. *IOP Conf. Ser. Mater. Sci. Eng.* **370**, 012001. 10.1088/1757-899X/370/1/012001 (2018).

[14] Krause, S. R. *Effect of Engine Intake-Air Humidity, Temperature, and Pressure on Exhaust Emissions* 710835 (SAE, 1971). 10.4271/710835.

[15] Martyr, A., Martyr, A. J. & Plint, M. A. *Engine Testing: Theory and Practice* 3. ed. (Butterworth-Heinemann, 2007).

[16] Martyr, A., Plint, A. J. & Alexander, M. *Engine Testing, Third Edition 2007* (Butterworth-Heinemann, Berlingtom, MA, 2011). 10.1016/C2010-0-66322-X.

[17] Yu, X., Zhu, L., Wang, Y., Filev, D. & Yao, X. Internal combustion engine calibration using optimization algorithms. *Appl. Energy* **305**, 117894. 10.1016/j.apenergy.2021.117894 (2022).

[18] Atkinson, C. & Mott, G. Dynamic Model-Based Calibration Optimization: An Introduction and Application to Diesel Engines. 10.4271/2005-01-0026 (2005).

[19] Singh, S. et al. An overview of vehicular emission standards. *Mapan* **38**, 241–263. 10.1007/s12647-022-00555-4 (2023).

[20] Vossoughi, G. R. & Rezazadeh, S. Optimization of the calibration for an internal combustion engine management system using multi-objective genetic algorithms. In *2005 IEEE Congr. Evol. Comput.* Vol. 2, 1254–1261. 10.1109/CEC.2005.1554834 (2005).

[21] Hribernik, A. & Moskwa, J. J. Transient response of a cross-flow charge air intercooler and its influence on engine operation. *J. Dyn. Syst. Meas. Control.* **122**, 483–489. 10.1115/1.1286683 (2000).

[22] Ray, S., Vijaya Vittala, C. B., Chakraborty, T. K. & Bigya Metei, W. Cyclone as an intercooler: A preliminary experimental investigation. *Exp. Therm. Fluid Sci.* **26**, 417–419. 10.1016/S0894-1777(02)00167-X (2002).

[23] Nasution, H., Abdul Aziz, A., Abdul Latiff, Z. & Engkuah, S. comparison of air to air and air to water intercoolers in the cooling process of a turbocharger engine. *J. Teknol.*10.11113/jt.v74.4832 (2015).

[24] Wu, H., Dai, X. & Zhang, Y. Research of intercooler heat transfer based on CFD. *MATEC Web Conf.* **22**, 03024. 10.1051/matecconf/20152203024 (2015).

[25] Tang, X., Shi, Q., Li, Z., Xu, S. & Li, M. Research on the influence of the guide vane on the performances of intercooler based on the end-to-end predication model. *Int. J. Heat Mass Transf.* **192**, 122903. 10.1016/j.ijheatmasstransfer.2022.122903 (2022).

[26] Engkuah, S., Nasution, H. & Abdul Aziz, A. Performance characteristic study on air to water intercooler. *Appl. Mech. Mater.* **819**, 42–45. 10.4028/www.scientific.net/AMM.819.42 (2016).

[27] Oun, M., Farhat, S. & M. lrabeei,. The effect of turbocharger pressure and intercooler temperature on engine performance. *J. Eng. Res. Univ. Tripoli Libya* **23**, 103–116 (2017).

[28] Oun, M. S. The effect of turbocharger pressure and intercooler temperature on engine performance. *J. Eng. Res.*10.66411/jer.v23i.303 (2017).

[29] Effiom, S. O., Abam, F. I. & Nwankwojike, B. N. Cycle parametric study on the performance of aeroderivative gas turbine models developed from a high bypass turbofan engine. *Cogent Eng.* **4**, 1368115. 10.1080/23311916.2017.1368115 (2017).

[30] Liu, S. & Zhang, Y. Research on the integrated intercooler intake system of turbocharged diesel engine. *Int. J. Automot. Technol.* **21**, 339–349. 10.1007/s12239-020-0032-9 (2020).

[31] Nandakishora, Y., Khayum, N., Shaik, J. H. & Christydass, S. Hybrid solar-membrane cryogenic CO2 separation for coal power plants: An energy and sustainability perspective. *J. Renew. Sustain. Energy* **17**(4), 046305 (2025).

[32] Arif, S., Qadeer, A., Taweekun, J., Noranai, Z. & Kalvin, R. Effect of refrigeration assisted intercooler turbocharging on engine’s horse power. *J. Adv. Res. Fluid Mech. Therm. Sci.* **79**, 95–111. 10.37934/arfmts.79.2.95111 (2021).

[33] Mifdal, M. Y. A., Nuraida, M. H., Norzalina, O. & Shamil, A. H. Turbo intercooler cooling system. *IJESI* **4**, 49–56 (2015).

[34] Ma’arof, M. I. N., Chala, G. T., Suresh, S. & Suresh, S. The development of an aftermarket intercooler spray for turbocharged vehicles using ethylene glycol and hyaluronic acid. *IOP Conf. Ser. Mater. Sci. Eng.* **863**, 012066. 10.1088/1757-899X/863/1/012066 (2020).

[35] Liu, S., Tu, A., Li, Y. & Zhu, D. Optimization design and comparative analysis of enhanced heat transfer and anti-vibration performance of twisted-elliptic-tube heat exchanger: A case study. *Energies* **16**, 6336. 10.3390/en16176336 (2023).

[36] Burgold, S., Galland, J.-P., Ferlay, B. & Odillard, L. Modular water charge air cooling for combustion engines. *MTZ Worldw.* **73**, 26–31. 10.1007/s38313-012-0237-z (2012).

[37] Trzesniowski, M. *Powertrain* (Springer Fachmedien Wiesbaden, 2023). 10.1007/978-3-658-39885-9.

[38] Grönman, A., Sallinen, P., Honkatukia, J., Backman, J. & Uusitalo, A. Design and experiments of two-stage intercooled electrically assisted turbocharger. *Energy Convers. Manag.* **111**, 115–124. 10.1016/j.enconman.2015.12.055 (2016).

[39] Jiang, H., Wang, H., Jiang, F., Hu, J. & Hu, L. Research on the optimization of a diesel engine intercooler structure based on numerical simulation. *Processes* **12**, 276. 10.3390/pr12020276 (2024).

[40] Bang, H., Park, G. & Lee, S. Performance improvement of intercooler in biogas generators by using computational fluid dynamics. *Appl. Therm. Eng.* **243**, 122541. 10.1016/j.applthermaleng.2024.122541 (2024).

[41] Watson, N. & Janota, M. S. Introduction to turbocharging and turbochargers. In *Turbocharging Intern. Combust. Engine* 1–18 (Macmillan Education UK, 1982). 10.1007/978-1-349-04024-7_1.

[42] Arava, T., Ionescu, R., Miron, L. & Chiriac, R. An assessment of the operation under different ambient conditions of the charge-air cooler for a large marine diesel engine. *J. Mar. Sci. Eng.* **14**(9), 845 (2026).

[43] Serrano, J. R., De la Morena, J., Gomez-Vilanova, A. & Gonzalez-Dominguez, D. Evaluation of two-stage advanced turbocharging systems for medium duty hydrogen engine conversion: A comprehensive 1D engine modelling approach. *Fuel* **1**(395), 135152 (2025).

[44] Engine Power Test Code Committee, Engine Power Test Code - Spark Ignition and Compression Ignition - As Installed Net Power Rating, (n.d.). 10.4271/J1349_201109.

[45] Kaul, S., Joshi, T. & Varshney, L. Analysis of hot air duct between turbocharger and intercooler. *Mater. Today Proc.* **43**, 719–725. 10.1016/j.matpr.2020.12.846 (2021).

[46] Kapse, R. D., Arakerimath, R. R., G.H. Raisoni College of Engineering Management. Study and comparison of charge air cooling techniques their effects on efficiency of automobile engine. *Int. J. Eng. Res.* **V6**, IJERTV6IS070152. 10.17577/IJERTV6IS070152 (2017).

